# Merkel Cell Carcinoma from Molecular Pathology to Novel Therapies

**DOI:** 10.3390/ijms22126305

**Published:** 2021-06-11

**Authors:** Karolina Stachyra, Monika Dudzisz-Śledź, Elżbieta Bylina, Anna Szumera-Ciećkiewicz, Mateusz J. Spałek, Ewa Bartnik, Piotr Rutkowski, Anna M. Czarnecka

**Affiliations:** 1 Department of Soft Tissue/Bone Sarcoma and Melanoma, Maria Sklodowska-Curie National Research Institute of Oncology, 02-781 Warsaw, Poland; kk.stachyra@gmail.com (K.S.); Monika.Dudzisz-Sledz@pib-nio.pl (M.D.-Ś.); Elzbieta.Bylina@pib-nio.pl (E.B.); mateusz@spalek.co (M.J.S.); Piotr.Rutkowski@pib-nio.pl (P.R.); 2 Faculty of Medicine, Medical University of Warsaw, 02-091 Warsaw, Poland; 3 Department of Clinical Trials, Maria Sklodowska-Curie National Research Institute of Oncology, 02-781 Warsaw, Poland; 4 Department of Pathology and Laboratory Diagnostics, Maria Sklodowska-Curie National Research Institute of Oncology, 02-781 Warsaw, Poland; Anna.Szumera-Cieckiewicz@pib-nio.pl; 5 Department of Diagnostic Hematology, Institute of Hematology and Transfusion Medicine, 00-791 Warsaw, Poland; 6 Institute of Genetics and Biotechnology, Faculty of Biology, University of Warsaw, 02-106 Warsaw, Poland; ewambartnik@gmail.com; 7 Department of Experimental Pharmacology, Mossakowski Medical Research Centre, Polish Academy of Sciences, 02-106 Warsaw, Poland

**Keywords:** Merkel cell carcinoma, tumor mutational burden, immunotherapy, TP53, polyomavirus

## Abstract

Merkel cell carcinoma (MCC) is an uncommon and highly aggressive skin cancer. It develops mostly within chronically sun-exposed areas of the skin. MCPyV is detected in 60–80% of MCC cases as integrated within the genome and is considered a major risk factor for MCC. Viral negative MCCs have a high mutation burden with a UV damage signature. Aberrations occur in *RB1*, *TP53*, and *NOTCH* genes as well as in the PI3K-AKT-mTOR pathway. MCC is highly immunogenic, but MCC cells are known to evade the host’s immune response. Despite the characteristic immunohistological profile of MCC, the diagnosis is challenging, and it should be confirmed by an experienced pathologist. Sentinel lymph node biopsy is considered the most reliable staging tool to identify subclinical nodal disease. Subclinical node metastases are present in about 30–50% of patients with primary MCC. The basis of MCC treatment is surgical excision. MCC is highly radiosensitive. It becomes chemoresistant within a few months. MCC is prone to recurrence. The outcomes in patients with metastatic disease are poor, with a historical 5-year survival of 13.5%. The median progression-free survival is 3–5 months, and the median overall survival is ten months. Currently, immunotherapy has become a standard of care first-line therapy for advanced MCC.

## 1. Introduction

Merkel cell carcinoma (MCC) is an uncommon and highly aggressive skin cancer developing within the dermis and subcutis. It has an immunophenotype (Cytokeratin 20—CK20) corresponding to sensory Merkel cells of the skin—mechanoreceptor cells of the basal layer of the epidermis. More detailed analysis accumulating over the last years suggests that MCC cells (CK20+, CD56+, CKAE1/AE3+, NSE+, PAX5+, NSP+, TdT+, SATB2+, TTF-1−, LCA−, S100−, p40−, chromogranin+/−, and synaptophysin+/−) are not the progeny of mature Merkel cells (Cytokeratin 20—CK20+), and the true origin of MCC remains unknown [1]. There are a few hypotheses about the cellular origin of MCC, including deriving from epidermal and dermal stem cells, dermal fibroblasts, as well as pre/pro B cells [2]. MCC incidence rate is estimated at 0.25–0.32 per 100,000 persons annually, reaching 9.8 per 100,000 persons annually among people older than 85 years. The incidence is increasing steeply, most likely due to the aging population, improved recognition, and rising awareness of MCC [3]. The 10-year survival rate is estimated to be around 57% [4,5]. The prevalence in men is 1.5 times higher than in women. It develops within chronically sun-exposed areas of the skin and mostly affects the fair-skinned population at an advanced age. Around 30–50% of MCC cases occur within the skin of the head, while other common sites are upper and lower extremities [4,6]. It is very rarely detected within oral or genital mucous membranes [7]. Immunosuppression, excessive UV exposure, and Merkel cell polyomavirus (MCPyV) infection are the greatest risk factors for MCC. In fact, MCC incidence is rising over the last years due to the aging of the population, increased sun exposure, and increased use of immunosuppressive medications [8]. Immunological disorders such as AIDS or hematological malignancies such as chronic lymphatic leukemia, as well as solid-organ transplantations and UV light, result in the impairment of the immune response, which plays a particularly important role in MCPyV+ MCC development. In the process of non-viral-mediated carcinogenesis, ultraviolet radiation damages DNA [9,10,11,12]. MCPyV- MCCs contain numerous DNA mutations caused by UV damage, whereas MCPyV+ MCCs have an incorporated viral genome and few mutations with “UV signature”. MCPyV- MCCs have between a 25- and 90-fold increased rate of UV-induced mutations compared to MCPyV+ MCCs. Moreover, in the viral-mediated mechanism, the majority of genes are intact, and interaction between these wild-type genes and viral proteins results in the development of MCCs [9,13,14]. Additionally, the MCPyV+ MCC patients have a three-fold higher 5-year survival rate than MCPyV- MCC ones [15]. The median mutation burden in MCPyV- MCCs is estimated to be 1121 somatic single nucleotide variants (SSNVs) per exome with frequently downregulated *RB1* and *TP53*. Mutations affect genes responsible for chromatin modifications (*ASXL1*, *MLL2/3*) and DNA damage repair (*ATM*, *MSH2*, *BRCA2*). Aberrations of x-Jun N-terminal kinases (JNKs) (*MAP3K1, TRAF7*) were also identified. Activation of the PI3K pathway and suppression of the NOTCH pathway are present in MCCs [14]. Virus-positive and virus-negative MCC of unknown primary origin (MCC-UP) exhibit an immune profile similar to virus-positive and virus-negative primary cutaneous MCCs. Both virus-positive and -negative MCC-UP are immunogenic with high intratumoral PD-L1 expression as well as intratumoral CD8 and FoxP3 infiltrates. Moreover, MCPyV-negative MCC-UP tumors present typical UV signatures and high tumor mutational burden, and in contrast, low numbers of mutations are found in MCPyV-positive MCC-UPs [16].

**Figure 1:**
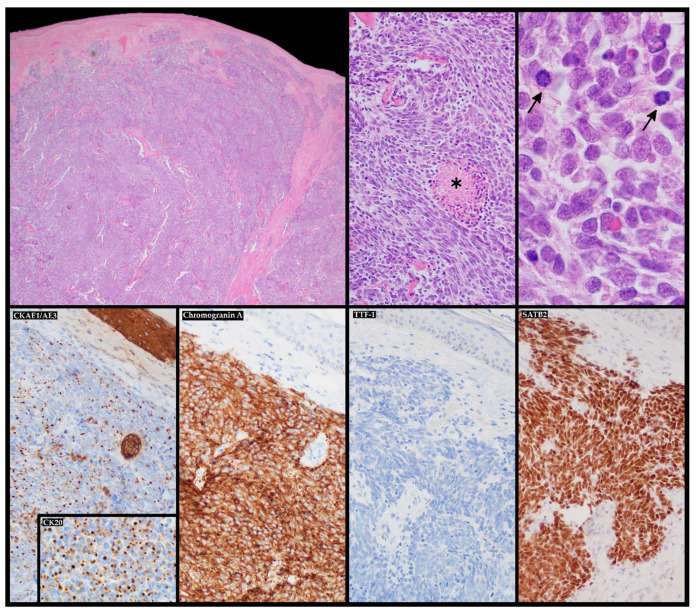
Merkel cell carcinoma histological features with its typical immunohistochemical profile (CK20+, CKAE1/AE3+, Chromogranin A+, SATB2+, and TTF-1-), focal necrosis (see: asterisk), and brisk mitotic activity (see: arrows). CK20—Keratin 20; CKAE1/AE3—Cytokeratin AE1/AE3—keratin cocktail that detects CK1—8, 10, 14–16 and 19, but does not detect CK17 or CK18; SATB2 Special AT-rich sequence-binding protein 2; TTF1—NK2 homeobox 1, also known as thyroid transcription factor 1.

**Figure 2:**
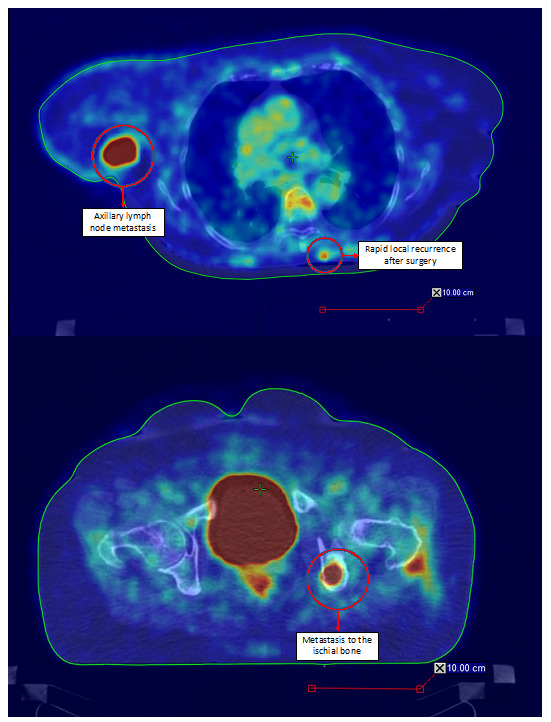
Positron emission tomography/computed tomography performed before adjuvant radiotherapy after surgery of Merkel cell carcinoma of the trunk showed the presence of rapid local recurrence (single lesion), single axillary lymph node metastasis, and single metastasis to the ischial bone.

The diagnostic clues for identification of MCC can be described by the AEIOU acronym (A—asymptomatic; E—expanding rapidly; I—immune-suppressed; O—older than 50 years; U—UV-exposed skin). While around 90% of MCC patients fulfill at least three criteria, only in 7% of them are all five detected. MCC is known to be solitary, and it is characterized as a firm, flesh-colored, or red-violaceous nodule with a shiny and smooth surface. Telangiectasia could also be observed. Merkel cell carcinoma can be clinically indistinguishable from other cutaneous malignancies, such as amelanotic melanoma, squamous cell carcinoma, or basal cell carcinoma [7,17,18]. Nevertheless, the histopathological review from the excisional biopsy is essential to confirm MCC diagnosis. In the microscopic examination, MCC manifests as expansile, nodular, or diffusely infiltrative lesions. Most MCC cases display neuroendocrine morphology. It consists of small blue cells with round or oval nuclei. There are plentiful active mitoses and apoptotic bodies. The nuclear–cytoplasmic ratio is high, and cytoplasm is scant. Granular chromatin creates “salt and pepper” nuclei, which are characteristic of neuroendocrine tumors. Further diagnosis is based on immunohistochemical staining, which differentiates MCC cells (CK20+, CD56+, CKAE1/AE3+, NSE+, PAX5+, NSP+, TdT+, SATB2+, TTF-1−, LCA−, S100−, p40−, chromogranin+/−, and synaptophysin+/−) from other cancer cells such as basal cell carcinoma, metastatic neuroendocrine carcinoma, lymphoma, or Ewing sarcoma. Additionally, radiological examinations, including X-ray, computed tomography (CT), and magnetic resonance imaging (MRI), allow detecting local and distant metastases (Figure 1) [4,7,9,19]. The aforementioned types of examinations reveal crucial prognostic factors such as the size of the primary lesion, the presence of regional lymph node involvement, and distant metastases. This information is vital to assess the stage of the lesion according to the eighth edition of the American Joint Committee on Cancer (AJCC) TNM (tumor–node–metastases) criteria. The MCC stage determines further treatment and prognosis (Figure 2) [4,20]. Most recently, in radiology reports, it has been shown that positron emission tomography/computed tomography (PET/CT) is an effective imaging tool in metastatic or unresectable MCC. In most reports, the glucose analog ^18^F-fluorodeoxyglucose (^18^F-FDG) was used as a radiotracer, although ^68^Ga-labeled somatostatin analogs have been employed as well as 6-fluoro-(^18^F)-L-3,4-dihydroxyphenylalanine (^18^F-DOPA). Nevertheless, currently, PET-CT is still inferior in sensitivity in detecting the early stages of MCC in comparison to sentinel lymph node biopsy, but an important role for ^18^F-FDG PET/CT in the staging of MCC is expected [21,22].

## 2. Merkel Cell Polyomavirus

Within the polyomaviridae family of nonenveloped, icosahedral, double-stranded DNA viruses, 13 human polyomavirus (HPyVs) species have been discovered. HPyVs affect numerous human tissues such as the respiratory system, kidney, or skin, often causing asymptomatic, persistent infection. Nevertheless, they become active due to impairment of the immune system, resulting in severe diseases, including respiratory infections, allograft interstitial nephritis, hemorrhagic cystitis, multifocal leukoencephalopathy, and cancers. The risk of developing these diseases is particularly high in immunosuppressed patients after transplantation as well as after HIV infection [23,24].

Merkel cell polyomavirus (MCPyV) infection is ubiquitous amongst humans and is usually acquired in early childhood. No route of MCPyV transmission is validated, but saliva and/or skin-to-skin close contact, as well as the fecal–oral transmission route, are suggested. MCPyV was proven to have widespread tissue distribution with higher levels of MCPyV in the upper aerodigestive tract and digestive system in comparison to the genitourinary system or lungs. About 15% of healthy individuals have MCPyV DNA on their skin. It mainly enters keratinocytes and dermal fibroblasts. Seroprevalence for MCPyV depends on the examined population, but it presents a similar trend to increase with age with an estimated 53–96.2% overall MCPyV seroprevalence [25,26,27,28,29,30,31,32,33,34,35]. Once acquired, MCPyV becomes a life-long resident of human skin flora [35]. The copy number of integrated viral genomes varies from cell to cell, with a median of three viral copies per cell. Patients with a viral load higher than one copy per cell have better overall survival than MCPyV- ones. Detection of the virus in the blood is associated with worse outcomes [36,37]. Initially, MCPyV is latent, nonreplicative after infection, and detected in 60–80% of MCC cases as integrated within the genome [38,39].

**Figure 3:**
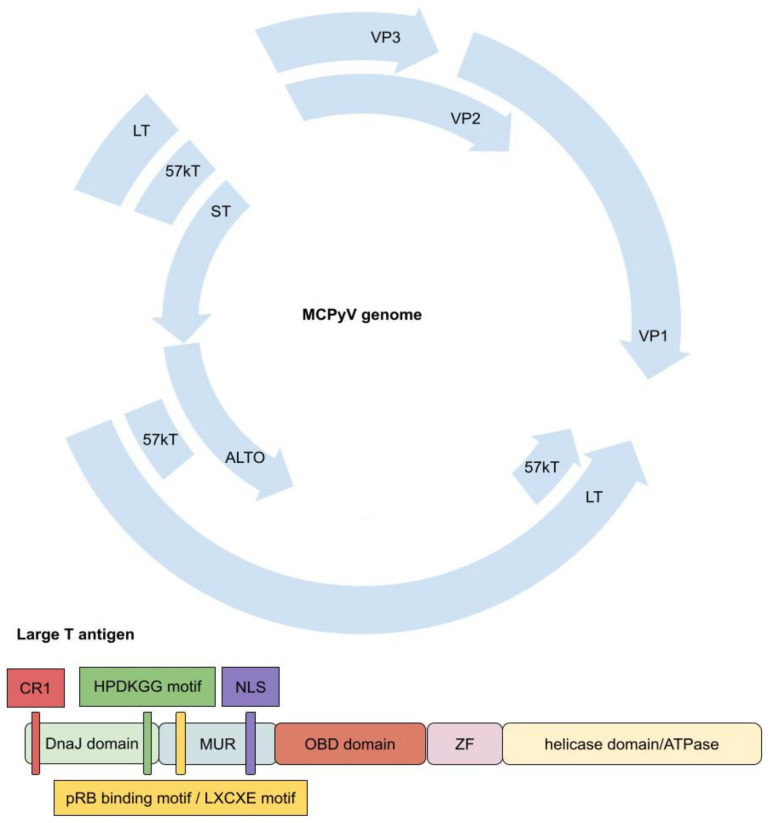
MCPyV genome and large T antigen structure. MCPyV genome consists of early and late regions. The late region comprises viral coat proteins VP1 and VP2, while the presence of VP3 is questionable. The early region encodes large T antigen (LT) and small T antigen (ST). When the early region undergoes alternative splicing, it produces 57kT. Moreover, in the process of the alternative translation initiation, an alternative frame of the large T open reading frame (ALTO) is expressed. Large T antigen consists of an N-terminal DnaJ domain, an MCPyV unique region (MUR), a DNA origin binding domain (OBD), the zinc finger domain (ZF), and a helicase domain/ATPase. The N-terminal DnaJ domain comprises a CR1 (conserved region 1) motif and an HPDKGG motif. MUR contains an LXCXE motif (pRB binding motif) and a nuclear localization signal (NLS).

MCPyV has a circular, double-stranded DNA genome of 5387 base pairs. It is divided into the bidirectional early and late regions, which are separated by a non-coding regulatory region (NCRR) containing the viral replication origin. NCRR comprises an AT-rich tract, LT-binding domain, and early enhancer region. The late region consists of viral coat proteins VP1 and VP2, while the presence of VP3 is questionable, as well as miRNA, which acts on T antigen transcripts. The early region encodes large T antigen (LT) and small T antigen (ST) as well as 57kT. Moreover, in the process of the alternative translation initiation, an alternative frame of the large T open reading frame (ALTO) is expressed. While the functions of 57kT and ALTO have not been identified, LT and ST play an important role in viral replication [9,36,40,41]. LT consists of an N-terminal DnaJ domain, an MCPyV unique region (MUR), a DNA origin binding domain (OBD), the zinc finger domain (ZF), and a helicase domain/ATPase. The N-terminal DnaJ domain comprises a CR1 (conserved region 1) motif and an HPDKGG motif that binds Hsc70. MUR contains an LXCXE motif (pRB binding motif) and a nuclear localization signal (NLS), and it binds to Vam6p, redistributing it to the nucleus. Moreover, the pRB binding motif presents a complementary sequence for an MCPyV viral miRNA. The C-terminal region of LT is thought to bind p53 by interaction with some bridging protein or one that changes LT conformation [42,43]. LT is responsible for viral replication not only by having a C-terminal DNA origin binding domain, but also by acting like a helicase, unwinding viral DNA. Additionally, it mobilises host cellular DNA polymerases [9,36,40,41,43]. ST shares a DnaJ domain with LT and at its C-terminal region contains two zinc-binding domains (CXCXXC motif), which are responsible for ST stability, and two domains rich in cysteine and proline, forming a protein phosphatase 2A (PP2A) binding site. ST is essential for stabilizing LT by its LT stabilizing domain (LSD), increasing the effectiveness of the viral replication process (Figure 3) [9,36,40,41,43].

Attachment of MCPyV requires sulfated polysaccharides like heparan sulfates and/or chondroitin sulfates and sialic acid as a coreceptor. It is internalized in small endocytic pits and enters the process of caveolar/lipid raft-mediated endocytosis. Microtubular transport, acidification of endosomes, and redox environment are essential in the trafficking. A minority of viruses are transported to the endoplasmic reticulum, while others remain at endosomal compartments. It is suggested that endosome-to-ER trafficking is one of the most significant limitations in MCPyV infection [44,45]. MCPyV DNA integration within the host genome displays a clonal pattern; thus, it is suggested that MCPyV infection precedes clonal expansion of MCC cells [39,46]. MCPyV infection is facilitated by matrix metalloproteinases (MMP), especially MMP1, MMP3, and MMP10, which degrade the extracellular matrix of the cell, providing MCPyV easier access to its surface. The induction of MMP expression is stimulated by epidermal growth factor (EGF), fibroblast growth factor (FGF), and inhibitors of glycogen synthase kinase 3 (GSK3), inducing WNT/β-catenin signaling. The WNT signaling pathway is also increased by classical clinical MCC risk factors, such as UV radiation and aging, which in this way facilitate MCPyV infection [1,32,40].

Viral integration into the human genome is reported within most chromosomes across genes. The site of MCPyV integration is unique for every tumor and thereby determines its further development. Nevertheless, due to the lack of integrase, integration is not a part of the MCPyV natural cell cycle. At the sites of microhomology between the host and MCPyV genomes, the mechanism of erroneous DNA repair is observed. It enables the virus to integrate into the human genome, mainly into transcriptionally active gene-dense regions and accessible chromatin as well as near short interspersed nuclear elements (SINEs), B double prime one, and a subunit of RNA polymerase III transcription initiation factor IIIB (BDP1) binding sites. Moreover, MCPyV tropism toward sensory perception and G-protein coupled receptor genes was suggested [46,47,48]. After infection, MCPyV enters in viral latency, which means that its genome does not replicate independently and it does not produce any infectious particles. Thus, a wide array of MCPyV mechanisms promotes this state. Highly phosphorylated LT protein initiates MCPyV genome replication by binding to the DNA replication origin. LT expression is suppressed by the autoinhibition of its own promoter in a negative feedback loop [49]. Additionally, the interaction between LT and RB1 results in activation of p14(ARF), which increases the cellular level of p53 [43]. Moreover, MCPyV encodes MCV-miR-M1-3p and MCV-miR-M1-5p single miRNAs, which are located on the antisense strand of the LT. MCV-miR-M1 displays a sequence complementary to the LXCXE motif in the LT mRNA transcript, repressing it. Additionally, MCV-miR-M1 targets antiviral protein SP100, causing decreased secretion of CXCL8 in consequence, leading to neutrophil chemotaxis depletion. The host immune evasion promotes persistent, long-term infection of MCPyV [50]. Additionally, LT phosphorylation sites are recognized by Skp-Celli-F box (SCF) E3 ubiquitin ligases F-box/WD repeat-containing protein 7 (Fbw7), F-box/WD repeat-containing protein 1A (βTrCP), and S-phase kinase-associated protein 2 (Skp2), which results in degradation of LT and preserves its low cellular level. Moreover, adverse cellular conditions, such as nutrient starvation, which inhibit the mechanistic target of rapamycin (mTOR) kinase, LT mutations in phosphorylation sites, or downregulation of SCF E3 ubiquitin ligases lead to accumulation of LT proteins and replication of MCPyV and initiate spreading to uninfected cells or to a new host [49].

In fact, oncogenic transformation by MCPyV requires two molecular events: integration of the MCP genome into the human genome and truncation of LT that makes the virus non-replicating. Mutations of MCPyV result in truncated LT (tLT). LT is truncated by premature stop codons or deletions that lead to loss of the C-terminal origin binding (OBD) and helicase domains, which are vital for MCPyV replication [36]. tLT preserves the pRB1-binding domain (LXCXE motif), which binds the cellular tumor suppressor retinoblastoma-associated protein (*RB1* gene) and thereby releases elongation factor 2 (EF2), promoting progression to the S phase of the cell cycle [9]. tLT also retains the DnaJ chaperone, which allows it to interact with Heat shock 70 kDa protein 8 (Hsc70), releasing EF2 from RB1 and stimulating MCPyV replication [51]. It also stimulates the expression of protein atonal homolog 1 (*ATOH1*) and SRY-box transcription factor 2 (*SOX2*). tLT is known to promote tumor growth more strongly than wild-type LT or 57kT [11]. Expression of the C-terminal 100 amino acids inhibits the growth of MCPyV+ MCC, but the mechanism is not known. It could be related to cell cycle checkpoint kinase, including ataxia telangiectasia mutated serine/threonine kinase (ATM), casein kinase 2β (CSNK2B), and phosphatidylinositol-5-phosphate 4-kinase type 2β (PIP4K2B), which alter cell proliferation by inhibiting progress from G1 to S cell cycle and promoting apoptosis [11]. Ubiquitin-specific protease 7 (Usp7) binds with all MCPyV T-antigens, including LT, tLT, and 57kT, and probably in an indirect way with ST, suppressing MCPyV replication [52]. Additionally, either LT or ST decreases tumor cell autophagy by increasing the expression of miRNAs, such as miR-30a-3p, miR-30a-5p, and miRNA-375. Those miRNAs have complementary sequences within mRNAs that take part in the production of autophagy proteins, such as autophagy-related 7 (ATG7), sequestosome 1 (SQSTM1,p62), and beclin 1 (BECN1). Interaction between miRNAs and mRNAs results in a decreased level of those autophagy proteins [53].

**Figure 4:**
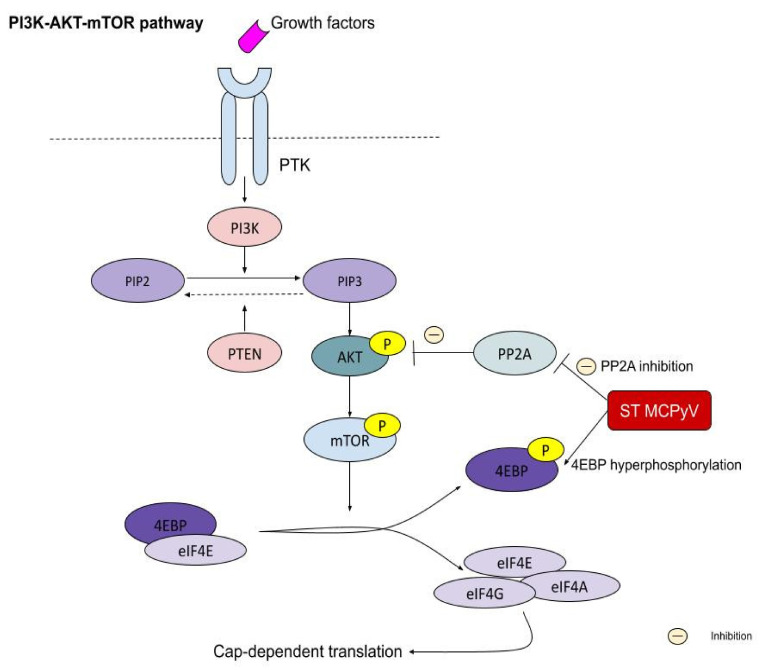
PI3K–AKT–mTOR pathway. ST MCPyV inhibits PP2A as well as promotes 4EBP hyperphosphorylation, resulting in increased frequency of cap-dependent translation. (PTK—protein tyrosine kinase, PI3K—phosphatidylinositol-3-kinase, PIP2—phosphatidylinositol 4,5-bisphosphate, PIP3—phosphatidylinositol 4,5-bisphosphate, PTEN—phosphatase and tensin homolog, AKT—AKT serine/threonine kinase, mTOR—mechanistic target of rapamycin kinase, PP2A—protein phosphatase 2A, 4EBP—4E binding protein, eIF4E/4G/4A—eukaryotic translation initiation factor 4E/4G/4A, ST MCPyV—small T antigen).

ST expression is required in the development of MCPyV+ MCC. ST protein plays a significant role in promoting mitogenesis and cell proliferation by upregulation of the PI3K-AKT-mTOR signaling pathway, which initiates cap-dependent translation (Figure 4). To promote this type of translation, eukaryotic translation initiation factor 4E (eIF4E) has to bind with eIF4A and eIF4G, creating multisubunit eIF4F. By mTOR phosphorylation of 4E-binding protein 1 (4E-BP1), it releases eIF4E, allowing it to start the cap-dependent translation. ST acts downstream in this pathway, leading to increased MCC growth by persistent hyperphosphorylation of 4E-BP1 [54]. Moreover, ST binds and inhibits protein phosphatase 2A (PP2A), thus maintaining AKT in a phosphorylated and active form. It also promotes the induction of the proglycolytic genes. ST inhibits particular E3 ubiquitin ligases, which normally take part in cell cycle regulation. It is known to promote centrosome overduplication (supernumerary centrosomes) and aneuploidy by targeting E3 ligases and on account of overlapping E3 ligase networks. It also induces the formation of micronuclei [55,56]. Additionally, the interaction between small T antigen and NF-κB essential modulator (NEMO) leads to the inhibition of IκB kinase α (IKKα)/IKKβ-mediated IκB phosphorylation, which regulates NF-κB heterodimer translocation to the nucleus [57]. ST binds to L-myc-1 proto-oncogene protein (MYCL), thereby regulating EP400 histone acetyltransferase and chromatin remodeling complex, which results in changed gene expression [38,58]. ST has an LT stabilizing domain (LSD), which increases the level of LT and enables it to disrupt the function of F-box/WD repeat-containing protein 7 (FBXW7) and cell division cycle protein 20 homolog (CDC20). The LSD domain plays a crucial role in MCPyV+ MCC development [9,11,36,38].

MCC patients are not contagious due to a tumor-related mutation in LT where the helicase domain is eliminated. tLT prevents autonomous replication of the viral genome, thus making MCPyV non-transmittable. Moreover, viral replication is inhibited by E3 ligase targeting of phosphorylated LT, downregulation of MCPyV promoter by LT, as well as underexpression of LT due to viral microRNA [38,49,59].

## 3. Immunogenicity of Merkel Cell Carcinoma

The association between increased Merkel cell carcinoma incidence and mortality rate and pathological immune dysfunction suggests a significant role of the immune system in MCC development. Approximately 10% of all MCC patients have decreased immunity. Alterations in the immune system are observed in the course of solid organ transplantation immunosuppression, chronic inflammatory conditions, HIV infection/AIDS, and hematological malignancies. A 30-fold higher MCC incidence is observed among patients with chronic lymphocytic leukemia [6,38,60,61,62,63,64]. Withdrawal of immune-suppressive treatment, as well as spontaneous regressions, are suggested to be linked with immune-cell mediated responses consisting of T cells and macrophages [65,66]. Ultraviolet radiation (UVR) also contributes to suppressed immunity due to the excess production of cytokine IL-10 and the generation of Tr1-like regulatory T cells. Both take part in the inhibition of Th1 and Th2 cells, which are essential for humoral immune response development [67].

As the cellular immune system is known to be pivotal in regulating MCC prevention and development, dense intratumoral infiltration, especially with CD8 T cells, is associated with a favorable prognosis [68,69,70]. MCPyV-specific CD4 and CD8 T-cell epitopes are observed in tumors and blood from MCPyV+ MCC patients. A higher number of MCPyV-specific T cells is observed within the tumor environment than in the blood. T cells could identify LT and ST oncoproteins as well as capsid structural protein antigens [71]. The MCPyV VP1 capsid protein was reported to not be expressed by MCPyV+ MCC, resulting in the lack of VP1-related responses in the tumor-infiltrated lymphocytes (TILs). However, anti-VP1 T cells are identified in peripheral blood lymphocytes [33,72]. In LT- and ST-directed responses, there is no difference between intratumoral and peripheral lymphocytes. While VP1 antigen-specific T cells are detected in both MCPyV+ MCC patients and the healthy MCPyV+ population, T-cell responses to the LT and ST antigens are observed only in MCPyV+ MCC patients. Additionally, LT and ST antigen-specific T cells were detected to be present even three years after removal of the primary lesion, which could be explained by the appearance of memory T-cell response [73,74]. Intratumoral TCR clonality could be interpreted as a marker of cell-antigen variety. While the intratumoral TCR repertoire in MCPyV+ MCC is clonal, MCPyV- MCC is known to have more diverse TCR due to its higher mutational burden. This phenomenon results from altered antigen expression caused by the presence of gene mutations. Those neoantigens are mostly patient-specific, and they could be used for future personalized treatment. Nevertheless, some data show that MCPyV+ MCC could have greater immunogenicity than virus-negative MCC, despite its higher mutational burden and neoantigen expression [14,74,75,76]. CD4 T cells, also known as T helpers, have a diverse impact on the immune system. They play an essential role in immune system stimulation by releasing cytokines and mediators, and they are also required for activation of CD8 T cells, increasing cross-presentation of antigen-presenting cells, and enhancing phagocyte function. CD4 Th1 cells produce interferon-γ (INF-γ), which is an important antiviral cytokine, while CD4 Th2 cells are the source of IL-10, an anti-inflammatory cytokine that suppresses Th1 and NK cells, as well as IL-13, which acts either as a promoter or inhibitor of tumor development [73]. Moreover, they are responsible for the stimulation of B cell antibody class switching. One study found that specific CD4 T cells with diverse receptor sequences were identified in 76% of assessed MCCs, and their concentration was 250-fold higher within the tumor environment than in the peripheral blood. The frequency of CD4 T cells is much lower in comparison with CD8 ones; thus, research concerning tumor-specific CD4 T cells is more difficult. Nevertheless, identifying MCC-specific CD4 T-cell epitopes enables the development of CD4-targeted therapies besides CD8-based treatment [71,77]. The presence of tumor-associated neutrophil infiltration results in a longer recurrence-free period and increased overall survival. Additionally, tertiary lymphoid structures (TLS) located near MCC also correlate with prolonged recurrence-free survival due to exceeded CD8/CD4 ratio in the tumor periphery, but not in its center [78].

Seroprevalence for MCPyV depends on the examined population, but it presents a similar trend to increase with age, with an estimated 53–96.2% overall MCPyV seroprevalence [29,30,31,33,34]. As observed in the cellular immune response, anti-VP1 antibodies are present in both MCPyV+ MCC patients and the healthy MCPyV+ population, while MCPyV large and small tumor-associated antigen antibodies are produced exclusively by MCPyV + MCC patients. Thus, screening of anti-LT and anti-ST oncoprotein antibody levels is useful in the disease progression and recurrence monitoring when anti-VP1 antibody titer could be a prognostic marker at the baseline. MCPyV+ MCC patients with high antibody titers are thought to have a better progression-free survival, but there is no association with any tumor characteristics [72,76,79,80,81,82].

**Figure 5:**
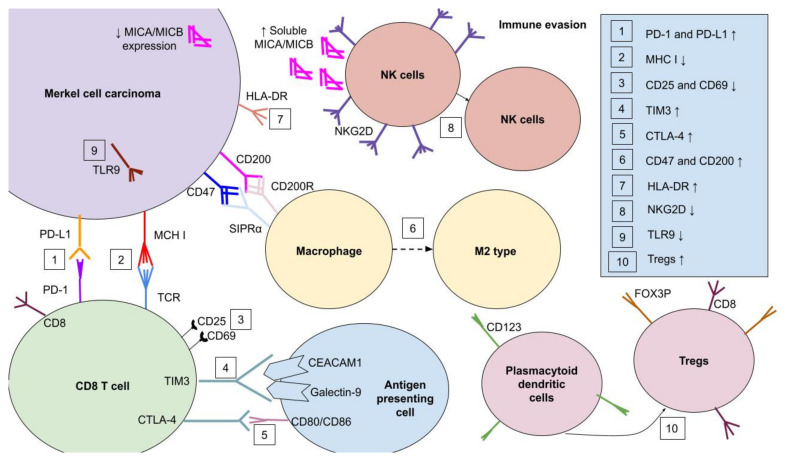
Merkel cell carcinoma immune evasion. To evade host immune response, MCC cells increase PD-L1, CD47, CD200, and HLA-DR expression. Boosted PD-1/PD-L1, CTLA-4/CD80, or CD86 and TIM3/CEACAM1 or galectin-9 interactions as well as decreased MCH1/TCR one result in immunosuppression. Overexpression of CD47 and CD200, promoting M2 macrophage polarization. In MCC cells, MICA/MICB and TLR9 expression is downregulated. Higher soluble MICA/MICB concentration causes NKG2D internalization and suppresses NK cell activity. Reduced expression of *CD69* and *CD25,* which are the markers of T-cell activation, is observed. Moreover, the proliferation of CD8+FOX3P+ Tregs is promoted by CD123+ plasmacytoid dendritic cells, which are located within the MCC environment.

Merkel cell carcinoma immune evasion is the process that impairs the patients’ immune system, facilitating MCC progression (Figure 5). One of those mechanisms is altered *HLA I* expression, resulting from genomic modifications or transcriptional regulation. In the former one, mutations or loss-of-heterozygosity in *HLA* genes are observed, while transcriptional suppression is mostly based on epigenetic alterations, such as hypermethylation, and is reversible in the ex vivo cultures by both interferon-γ (INF-γ) and the hypomethylating agent 5-azacytidine. INF-γ binds with the IFN receptor, which phosphorylates Janus kinase 1 and 2 as well as the signal transducer and activator of transcription protein 1 (JAK1/JAK2/STAT1), activating them. STAT1 stimulates interferon regulatory factor 1 (*IRF-1*) expression, which in turn upregulates *MHC I* expression [83]. To avoid immune resistance to cellular immunotherapy resulting from loss of particular *HLA* expression, the patient requires T-cell treatment, which is targeted to alternate HLAs whose expression is not decreased. Acquired immunotherapy resistance in the mechanism of transcriptional HLA downregulation is the consequence of MCC therapy restricted to a particular HLA T cell. It is also potentially reversible with drug treatment [84]. Antigen-specific T-cell responses are restricted only by a few HLA contexts, which could be caused by transcriptional loss of *HLA* expression on account of specific T-cell pressure [74]. Even when intratumoral CD8 T cells are present, they often show an exhausted phenotype that suppresses the immune response. It is characterized by a reduced expression of *CD69* and *CD25*, which are the markers of early and late T-cell activation, respectively. Moreover, exhausted T cells have upregulated expression of programmed cell death 1 (*PD-1*), T-cell immunoglobulin and mucin-domain containing-3 (*TIM3*), and cytotoxic T-lymphocyte associated protein 4 (*CTLA-4*), whose functions are described below. After in vitro treatment of MCC with IL-2 and IL-15, increased production of various cytokines, such as IFNγ, TNFα, IL-17, IL-10, IL-4, and IL-13, was observed, which enhances immune system activity. Additionally, repertoire skewing of TCR expression and upregulation of *CD137* were also identified. CD137 increases T-cell proliferation, cytolytic activity, as well as IL-2 production [73,85]. Another sign of immune avoidance is the enhanced number of suppressive CD25+FOX3P+ Tregs with the downregulation/lack of activated CD25+FOXP- T cells within the tumor environment. Moreover, CD4 and CD8 CD25+FOX3P+ Tregs increase *CTLA-4*, *GITR,* and *HLA-DR* expression, escalating immunosuppression. HLA-DR is thought to have high suppressive capacity. The proliferation of CD8+FOX3P+ Tregs is promoted by CD123+ plasmacytoid dendritic cells, which are located within the MCC environment [73]. Immune evasion is supported by macrophages switching from the M1 phenotype during tumor initiation toward M2 macrophages within the MCC development process. Their polarization depends on transcription factors, including phosphorylated signal transducer and activator of transcription 1 (STAT1), recombination signal binding protein for immunoglobulin kappa J region (RBP-J), or MAF bZIP transcription factor (CMAF). M2 macrophage colonies could be stimulated by IL-4/IL-13, IL-1β/LPS with immune complex, and IL-10/TGFβ/glucocorticoids. While classically activated M1 macrophages, stimulated by INF-γ and LPS, contribute to the Th1 response, alternatively activated M2 macrophages promote Th2 cells as well as tissue repair. Overall, M2 macrophage dominance supports tumor progression [86,87]. MHC class I chain-related protein (MIC) A and B production is stimulated by either viral infection or malignancy. MIC is known to take part in cell stress transmission within the immune system. It eliminates target cells by using Natural Killer group 2D (NKG2D). NKG2D ligands, such as MIC, make altered cells more vulnerable to NK cell-dependent tumor lysis. Hypoacetylation of the *MIC* promoter leads to its epigenetic silencing, resulting in its decreased expression. Moreover, MIC proteolytic shedding from the tumor cell surface results in decreased lesion recognition of NK cells, and the expanding amount of soluble NKG2D ligands in the tumor microenvironment causes NKG2D internalization and suppresses NK cell activity. These phenomena could be observed in MCC patients, causing facilitated development of the tumor [88,89,90]. Toll-like receptor 9 (TLR-9) is stimulated by unmethylated cytosine–phosphate-guanine dinucleotides, which are located in bacterial as well as viral DNA. *TLR-9* is constantly expressed both in plasmacytoid dendritic cells and B cells. It is also identified in neutrophils, monocytes, and CD4 T cells. TLR-9 ligands contribute to anti-tumor immune response. To prevent TLR-9-mediated activation of immune cells and promote tumor growth, LT and ST MCC antigens silence expression of this receptor [91,92,93,94,95,96]. *CD47,* as well as *CD200* overexpression, is also detected in MCC patients. CD200R is located in human monocytes and macrophages, and its activation by CD200 promotes immunosuppressive M2 polarization with increased IL-10 production and Tregs induction. On the other hand, CD47 binds to SIPRα receptor on macrophages, preventing phagocytosis of altered cells [38,97,98].

### 3.1. PD-1/PD-L1 Signaling in MCC

One of the most important immune escape mechanisms is increased expression of *PD-1* either in tumor-infiltrating or peripheral antigen-specific T cells and upregulated *PD-L1* expression at the surface of tumor cells, intratumoral macrophages, and peritumoral immune cells. PD-L1 shares homology with B7.1 (CD80) and B7.2 (CD86) [78,99]. PD-1/PD-L1 signaling suppresses the immune response in peripheral tissues in order to prevent damage to normal unchanged cells. The PD-1 pathway is considered to suppress already activated T cells at the tissue sites. Normally, *PD-1L* expression is detected on leukocytes and other cells, including those that are located within nonlymphoid tissues. Upregulation of *PD-L1* is caused by inhibitory pathways, for instance resulting from IFN-γ interaction or tumor signaling. *PD-L2* is expressed mainly on dendritic cells and monocytes. Nevertheless, it is also presented on a diverse group of other cells [100,101,102,103]. The PD-1/PD-L1 pathway enables MCC to avoid the immune system, resulting in undisturbed expansion. Approximately 42–49% of MCCs, 55% of TILs, and 39% of peritumoral macrophages have a strong *PD-L1* expression. Neither age nor sex nor initial diagnosis was associated with PD-L1 and PD-1 number in cells. Moderate or high TIL infiltration correlates with 100% *PD-L1* expression by MCC cells. High *PD-L1* expression and brisk lymphocyte response were exclusively demonstrated by MCPyV+ MCC compared to MCPyV- MCC. Those findings suggest that tumor-specific, potentially MCPyV-antigens, increase tumor *PD-1L* expression; hence, virus-positive MCC is considered to have greater immunogenicity in comparison to virus-negative MCC due to better response to PD-1/PD-L1 blocking therapies. PD-1/PD-L1 signaling is a great therapeutic approach in the immune checkpoint inhibitor MCC therapy [75,78,99]. Moreover, PD-L1 and PD-L2, which are both detected in the MCC microenvironment, are expressed by CD11c+ infiltrating dendritic cells as well as a subpopulation of CD163+ macrophages, instead of being produced by tumor cells [73].

### 3.2. Other Immune Receptors in MCC

*OX40* (*CD134, TNFRSF4*) is a member of the tumor necrosis factor receptor (TNFR) superfamily. It is expressed both in activated CD4 and CD8 T cells as well as on Tregs. CD4 T cells are considered to have more persistent expression of *OX40*, while CD8 T cells are more likely to have transient expression. Neither most memory T cells nor naïve T cells have OX40 on their surface. TCR–MHC interaction, activation of other costimulatory receptors, including CD28, as well as the presence of proinflammatory cytokines, such as IL-1, IL-2, IL-4, or TNF, in the microenvironment lead to enhanced *OX40* expression. Moreover, OX40 contributes to the two stages of Tregs development. The first one is driven by TCR signals with additional OX40–OX40L co-stimulation, while the second process is TCR-independent and also requires IL-2 and OX40–OX40L interaction [104]. Conventionally, OX40L is present on antigen-presenting cells (APC), but it is also detected on NK cells, mast cells, vascular endothelial cells, and smooth muscle cells [105,106,107]. Its upregulation results from the presence of inflammatory cytokines, including GM-CSF, IL-1, IL-2, IL-12, IL-15, TNF, and thymic stromal lymphopoietin (TSLP), as well as by interaction with the NK cell receptor—NKG2D [108,109,110]. OX40 is expressed on T cells early after antigen activation, while upregulation of *OX40L* is observed in APC only within an acute inflammatory microenvironment [111]. OX40 and OX40L interaction enhances T-cell clonal expansion, effector differentiation, and survival, resulting in greater immune response. Increased cytokine secretion, such as IL-2, IL-4, IL-5, and IFN-γ by CD4 T cells, is also observed as a result of OX40 stimulation. Additionally, OX40-stimulated T cells with expression of BCL-2, BCL-XL, and survivin play an important role in clonal proliferation of memory T cells [112,113,114,115]. As MCC is known to evade the immune response, downregulation of *OX40* and *OX40L* could be observed. Thus, OX40 agonist treatment enhances anti-tumor immunity and could contribute to MCC regression [38,114,116].

Glucocorticoid-induced TNFR-related protein (*GITR*,*TNFRSF18*), as an *OX40*, is a member of the TNFR superfamily. GITR is expressed on T and NK cells, and it is activated by GITRL, which is identified on APC and endothelial cells. GITR was detected on CD8+FOX3P+ T cells and on CD4+ Tregs within the intratumoral environment of MCC [73]. Due to co-stimulation of T cells and NK cells, suppression of Tregs, and macrophages and DC activation, GITR–GITRL interaction leads to the development of immune response against infection and tumor [117]. The therapeutic targeting of GITR might inhibit Tregs while enhancing CD8+ T cells, hence providing the clinical benefits in MCC treatment [118,119].

CD28, which binds either to CD80 or CD86, results in increased proliferation, survival, and differentiation of T cells due to IL-2 expression, metabolism upregulation, and higher expression of cell survival genes [101]. CTLA-4 (CD152), a CD28 homolog, has opposed activity to CD28, being a negative regulator of T-cell proliferation and activity. As with other checkpoint pathways, it plays an essential role in preventing autoimmunity by supporting peripheral tolerance. CTLA-4 is thought to inhibit potentially autoreactive T cells during the initial stage of immune response development, especially in lymph nodes [100]. On account of *CTLA-4* expression in MCC, tumor cells are vulnerable to anti-CTLA-4 antibody treatment. Moreover, interaction between anti-CTLA-4 antibodies and CTLA-4 on tumor cells results in activation of the EGFR pathway, which leads to increased *PD-L1* expression. Upregulated *PD-L1* expression induces a greater response to anti-PD-1 treatment [120,121]. Moreover, the recombinant forms of CTLA-4 ligands cause tumor cell apoptosis by inducing caspase-8 and caspase-3 [122].

Besides PD-1, MCC-targeting T cells express significantly more T-cell immunoglobulin and mucin domain-containing protein 3 (*TIM3*) than normal ones. TIM3 is detected on CD4 and CD8 lymphocytes, and it is defined as a “co-receptor”. It plays a significant role in immune suppression after binding to its two ligands, galectin 9 and carcinoembryonic antigen-related cell adhesion molecule 1 (CEACAM1). Activation of TIM3 results in releasing HLA-B-associated transcript 3 (BAT3), and it also increases interaction with the tyrosine kinase FYN [123,124]. It also has an impact on CD45 and CD148 [125]. Hence, TIM3 leads to immune synapse disruption and phosphatase recruitment, as well as participating in apoptosis. It is suggested that TIM3 function depends on the presence of a main inhibitory signal; thus, due to the lack of suppressing motif, it might even act as a co-stimulatory receptor. Both *PD-1* and *TIM3* are co-expressed and upregulated on dysfunctional T cells; thus, co-blockade of TIM3 and PD-1 in MCC therapy is promising [85,126].

## 4. Mutational Tumor Burden

The mutational tumor burden of Merkel cell carcinoma varies significantly between virus-negative and virus-positive MCCs. MCPyV- MCCs have a higher overall mutational rate with the median of 1121 somatic single nucleotide variants (SSNVs) compared to 12.5 SSNVs in MCPyV+ MCCs. The number of SSNVs ranges from 3 to 4707 per exome [14]. Another study found 10.09 ± 2.32 mutations/Mb in MCPyV- tumors and 0.40 ± 0.09 mutations/Mb in MCPyV+ [13]. MCPyV- and MCPyV+ MCCs median numbers of neoantigens were estimated to be 173 and 7 SSNVs per exome, respectively. That is the reason why intratumoral TCR repertoire in MCPyV+ MCC is clonal while MCPyV- MCC is known to have more diverse TCR. UV-mediated tumorigenesis is represented by a specific type of mutation, which is C to T transitions. Enrichment of UV-induced mutations was detected in 66–85% of MCPyV- MCCs, while in MCPyV+ MCCs they were not identified. The typical mutational signature of virus-positive MCCs has not been investigated. Mutational burden, as well as the number of C to T transitions, does not correlate with the primary MCC site and the patient’s age [13,14]. Those observations indicate diverse mechanisms of different types of MCC development.

## 5. Deregulated Genes and Signaling Pathways

### 5.1. RB1 Gene

**Figure 6:**
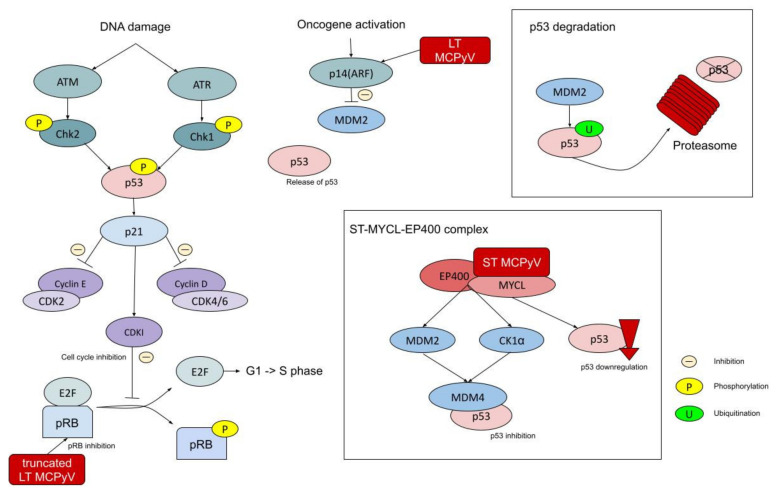
Alterations of pRB and p53 functioning in MCC. Truncated LT MCPyV inhibits pRB, causing E2F release and G1 to S phase transition. ST MCPyV binds to EP400 and MYCL, thus resulting in p53 downregulation as well as MDM4 overexpression by targeting MDM2 and CK1α. In contrast, LT MCPyV induces accumulation of p14(ARF), which inhibits MDM2, thereby releasing p53. (ATM—ATM serine/threonine kinase, ATR—ATR serine/threonine kinase, Chk1/Chk2—checkpoint kinase 1/2, CDK2—cyclin-dependent kinase 2, CDK4/6—cyclin-dependent kinase 4/6, CKDI—cyclin-dependent kinase inhibitor, MYCL—MYCL proto-oncogene, bHLH transcription factor, EP400—E1A binding protein p400, MDM2—MDM2 proto-oncogene, MDM4—MDM4 regulator of p53, CK1α—casein kinase 1α, LT—large T antigen, ST—small T antigen).

The *RB1* gene encodes a tumor suppressor protein called pRB, which acts by inhibiting cell cycle progression. pRB forms a complex with E2F, binding to its transactivation domain and maintaining the cell in the G1 phase. This complex binds to promoters of the genes essential for the transition to the S phase, repressing their transcription. pRB also remodels the chromatin structure by interaction with the proteins responsible for nucleosome remodeling, histone de-acetylation, and methylation, including SWItch/Sucrose Non-Fermentable (SWI/SNF) proteins, brahma-related gene-1 (BRG1), histone deacetylase 1 (HDAC1), DNA (cytosine-5)-methyltransferase 1 (DNMT1), and heterochromatin protein 1 (HP1) [127]. The copy loss of the *RB1* gene is detected in 45–63.5% of MCPyV- MCC cases; thus, LT-negative tumors are mostly also pRB-negative [13,14]. Additionally, there is a significant association between tumor *RB1* and viral *LT* antigen expression in MCPyV+ MCCs [128,129]. In MCPyV+ MCCs, a viral truncated large T antigen binds to pRB by the LXCXE motif—MCPyV-LT potently. Inactivation of pRB by MCPyV-LT is sufficient for MCC tumor growth out of MCPyV-positive MCC cells (Figure 6) [130]. Additional mechanisms indicated as inducing pRB downregulation in MCC are 13q deletions within the *RB1* locus, *RB1* promoter hypermethylation, truncating nonsense mutations, and miRNA-induced downregulation of expression [128,131,132]. Despite the difference in the mechanisms, in both viral and non-viral MCC, a lack of functional *RB1* results in an uncontrollable proliferation of the altered cells. This pathologic process underlies MCC carcinogenesis [9,13].

### 5.2. TP53 Gene

Tumor protein p53 (*TP53*), also known as the “guardian of the genome”, is a well-known tumor suppressor gene. It maps on the short arm of chromosome 17 (17p13.1). It is involved in cell cycle regulation, apoptosis induction, and DNA repair [133]. The frequency of *TP53* inactivating mutations has been estimated to be between 10% and 87.5% [13,14,134,135,136]. MCPyV- MCCs frequently contain inactivating mutations of *TP53* or its deletions, while in MCPyV+ MCCs, *TP53* was reported mostly not to be mutated, but its activity was downregulated [9,13]. In fact, both in MCPyV-negative and in MCPyV-positive cases, *TP53* inactivating mutations were reported [136]. Very recently, a complicated loop of molecular interactions was discovered in MCC pathogenesis. MCPyV LT that binds to pRB induces accumulation of p14(ARF) (cyclin-dependent kinase inhibitor 2A; p14) that physiologically is an inhibitor of MDM2 (p53 ubiquitin ligase). At the same time, expression of ST reduced p53 activity. MCPyV ST recruits *MYCL* proto-oncogene to the EP400 chromatin remodeling complex that decreases p53 expression—MCPyV complex inactivates p53. EP400 targets MDM2 and CK1α, activators of MDM4. As an effect, high levels of MDM4, which binds the p53 tumor suppressor protein and inhibits its activity, are detected (Figure 6) [137].

### 5.3. NOTCH Genes

In MCCs, mutations also occur in the NOTCH family, which can function as either oncogenic or suppressor genes, depending on the type of cell. Both NOTCH1 signaling, which takes part in cell fate determination, proliferation, differentiation, and apoptosis, and NOTCH2 signaling, which is responsible for the determination of the cell destiny in the embryo as well as for regulation of the immune system and tissue repair, are particularly affected in MCCs. A total of 75% of MCPyV-MCCs demonstrate *NOTCH* alterations, where *NOTCH1* mutations are identified in 50–96% of MCCs cases [138]. The majority of mutations contain a typical UV signature. *NOTCH* mutations are mostly identified within EGF and ankyrin repeat regions, which confirms the inactivating nature of these mutations. Loss-of-function mutations occurring in MCPyV-MCCs indicate their suppressive role in MCCs and other neuroendocrine malignancies [9,13,14]. It was shown that the expression of *NOTCH3* is an independent predictor of MCC outcome [139].

### 5.4. Hedgehog Signaling Pathway

The hedgehog (HH) signaling pathway regulates growth in embryos as well as enables maintenance of the stem/progenitor cell population and control of hair follicle and sebaceous gland development. HH signaling also controls cell proliferation by upregulation of *MYCN*, cyclin D, and E, as well as forkhead box protein M1 (*FOXM1*) [140]. It is activated by HH ligands including Sonic HH, Indian HH, and Desert HH as well as by alternative cascades triggered by V-Ki-Ras2 Kirsten rat sarcoma 2 viral oncogene homolog (KRAS), transforming growth factor-beta 1 (TGF-β), phosphatidylinositol-4,5-bisphosphate 3-kinase (PI3K), AKT serine/threonine kinase (protein kinase B, PKB), and protein kinase C alpha (PKC-α) that stimulate glioma-associated oncogene (GLI) zinc finger transcription factor (TF) [141]. In the conventional pathway, HH ligand binds to transmembrane receptors—protein patched homolog 1 (PTCH1) and protein patched homolog 2 (PTCH2), which sustain PTCH-SMO (Smoothened, Frizzled Family Receptor) inhibition. SMO transmembrane G protein-coupled receptor releases GLI proteins (GLI1/2/3) from their repressor—cytoplasmic suppressor of fused homolog 9 (SUFU) protein [142]. GLI TFs transmit the signal to the nucleus, and depending on its type (GLI1—activation, GLI2/3—activation or suppression), they regulate transcription of target genes, including cell cycle regulators such as *E2F*, *D-type cyclins*, *Cyclin A2*, *CDK1*, and *Cyclin B1*, as well as proto-oncogene int-1 homolog (*WNT*), *TGF-β*, forkhead transcription factor (*FOXE1*), zinc finger protein *SNAI1*, *PTCH1,* and itself—*GLI1*. GLI1 creates a positive feedback loop, whereas PTCH1, PTCH2, and hedgehog interacting protein (HIP) act as negative regulators [140,143,144,145,146,147]. Hedgehog pathway-related genes are upregulated in the majority of MCCs cases. Altered expression was observed in *Sonic HH* (93%), *PTCH* (86%), *GLI3* (86%), *Indian HH* (84%), *SMO* (79%), *GLI1,* and *GLI2* (79%) genes. Moreover, higher expression of *Sonic HH* was identified in virus-positive MCCs in comparison with virus-negative ones. Increased expression of *Sonic HH*, *Indian HH*, *PTCH,* and *GLI1* was significantly correlated with a better patient prognosis [148,149].

### 5.5. PI3K–AKT–mTOR Pathway

The PI3K–AKT–mTOR pathway is known to be overactivated in MCCs. Phosphatidylinositol 3-kinase (PI3K), whose p110α subunit is encoded by the *PIK3CA* gene, takes part in cell movement, growth, and proliferation as well as migration and translation of new proteins. Moreover, it phosphorylates signaling molecules that transmit signals from the cytoplasm to the cell nucleus and is involved in the regulation of insulin and maturation of fat cells. Thus, the PI3K/AKT pathway is essential for cell survival. PTEN acts as a negative regulator of PI3K and is a tumor suppressor, preventing the cell from growing and dividing uncontrollably. Allelic loss at 10q23 is associated with loss of the *PTEN* gene. Gain-of-function mutations in *AKT1*, *HRAS*, and *KRAS* have also been reported. Moreover, activating mutations occur in phosphatidylinositol-3,4,5-trisphosphate dependent Rac exchange factor 2 (*PREX2*), which is an inhibitor of PTEN [9,13,14,150].

### 5.6. Chromatin Modifying Genes

BMI-1 is a subunit of the polycomb repressor complex 1 (PRC1), which performs chromatin remodeling by histone 2A monoubiquitylation and its further methylation. It plays an important role in the regulation of morphogenesis in embryonic development and hematopoiesis during pre- and postnatal life, controlling the cell cycle as well as the DNA damage response [151]. *BMI-1* expression is altered in 78% of MCCs [152]. Enhancer of zeste homolog 2 (EZH2) is a member of the polycomb repressive complex 2 (PRC2). This complex methylates lysine 27 of histone H3, causing inhibition of gene transcription and heterochromatin formation [153]. In 54% of MCC, *EZH2* is overexpressed and results in downregulation of tumor suppressor genes, thus contributing to tumor progression. Higher expression of *EZH2* is associated with a poorer MCC patient’s prognosis [154,155]. Lysine methyltransferase 2D (*KMT2D*) and lysine methyltransferase 2C (*KMT2C*) encode mixed-lineage leukemia 2 (MLL2) and mixed-lineage leukemia 3 (MLL3), respectively. Both *KMT2D* and *KMT2C* are tumor suppressors and are downregulated in MCC. Moreover, *KMT2D* was demonstrated to be involved in MCPyV integration, which leads to the inactivation of this tumor suppressor in MCPyV+ MCC [14,46]. Additionally, sex combs like transcriptional regulator 1 (ASXL1), a member of polycomb group proteins, take part in the deubiquitylation of mono-ubiquitylated lysine 119 on histone H2A. *ASXL1* is a tumor-suppressor gene, so its lower expression in MCC results in inhibition of cell-protective INK4B-ARF-INK4A signaling, especially production of p53, and as a consequence of tumor progression [14,156].

### 5.7. Vascular Endothelial Growth Factors

Vascular endothelial growth factors (VEGFs) are endothelial cell-specific mitogens that are responsible for angiogenesis and lymphangiogenesis. Enhanced expression of intratumoral VEGFA, which promotes angiogenesis within the MCC environment, results in higher risk of metastases and is associated with poorer recurrence-free and overall survival [157,158,159]. *VEGFA* upregulation is suggested to be driven by MCPyV oncoproteins in virus-positive MCCs due to its significantly higher level than in MCPyV- MCCs. *VEGFA* is overexpressed in 91–95% of MCC cases [152,160]. Additionally, *VEGFC* is also upregulated in 75% of MCCs [152]. Vascular endothelial growth factor receptor-2 (*VEGFR-2*) expression in MCC correlates with tumor size as well as metastatic potential. *VEGFR-2* is upregulated in 91% of large (>2cm) MCCs and in 70% of small size (<2 cm) MCCs [161]. 

### 5.8. Tyrosine Kinase Receptors

*KIT* proto-oncogene encodes a receptor tyrosine kinase, which is a transmembrane receptor for stem cell factor (SCF) and is known to enhance the progression of human tumors [162]. *KIT* is expressed in 42.5–67% of MCCs, while its ligand is detected approximately in 8.6–31% of them. Co-expression of *KIT* and *SCF* is identified only in 16% of MCC cases. Four *KIT* point mutations were detected where two silent mutations were within exons 16 and 18 and two more were located in intron 16-17. *KIT* overexpression is associated with higher risk of progression and metastases. Platelet-derived growth factor receptor alpha (PDGFRA) is a surface tyrosine kinase receptor for the PDGF family. PDGFRA is responsible for wound healing, organ development, and tumor development, including MCCs. *PDGFRA* and *PDGFA* are overexpressed in 31.9–87% and 81% of MCCs, respectively. Co-expression of these molecules was detected in 81% of all MCC cases. Approximately 12.5% of MCC patients have common *PDGFRA* exon 10 S478P substitution [163,164,165]. Anaplastic lymphoma kinase (ALK) is also a receptor tyrosine kinase, occurring within many types of human tumors. ALK signaling exhibits anti-apoptotic function and also promotes proliferation, differentiation, and migration. *ALT* upregulation is observed in 51% of MCCs [154,166,167].

### 5.9. BCL-2 Family

The BCL-2 family is responsible for the regulation of apoptosis. BCL-2 itself is a pro-survival protein, such as BCL-XL and myeloid cell leukemia-1 (MCL-1). The ratio of anti-apoptotic and pro-apoptotic proteins determines whether a cell will survive or die [168]. *BCL-2* is considered a proto-oncogene, mainly due to its pro-survival effect. As normal Merkel cells, 80–85% of MCC cells also express *BCL-2* constitutively. There were no differences in MCPyV DNA between BCL-2 positive and negative MCC. BCL-2 negative tumors are more likely to metastasize and have lower overall survival compared to BCL-2 positive MCCs [169,170]. Anti-BCL-2 therapy alone was considered insufficient. Glaucarubin-induced DNA damage results in apoptosis of MCPyV+ MCC, promoting its regression. Thus, the combination of *BCL-2* inhibition and DNA damage-induced apoptosis constitutes a future treatment strategy for virus-positive MCCs [171]. MCL-1 sequesters pro-apoptotic BAK/BAX proteins, resulting in inhibition of apoptosis. It is altered in approximately 88% of MCC patients. MCL-1 targeting therapy may be a promising tumor treatment [152,172].

### 5.10. Toll-Like Receptors

Toll-like receptors (TLRs) play a significant role in the first line of the immune system. They are expressed in all innate immune cells and in cells of other tissues. TLRs recognize either exogenous or endogenous danger signs, pathogen-associated molecular patterns (PAMPs), and damage-associated molecular patterns (DAMPs), respectively. PAMPs are known to be microbial molecular structures, including pathogen-specific proteins, lipoteichoic acid, peptidoglycan from Gram-positive bacteria, and lipopolysaccharide of the Gram-negative bacteria outer membrane as well as RNA of viruses and DNA from viruses and bacteria. DAMPs are products of host cell injury or non-physiological death, such as extracellular matrix and plasma membrane components, cytosolic and nuclear proteins, and damaged elements of organelles [173]. *TLRs* altered expression is considered to reflect a clinicopathological hallmark as well as to be a prognostic marker of MCC. Downregulation of *TLR9*, which identifies unmethylated CpG DNA motifs, is significantly associated with virus-positive MCC. Nuclear expression of both *TLR2*, which is a receptor, e.g., for peptidoglycan and lipoarabinomannan, and *TLR5*, a receptor for flagellin, correlates with large tumor size, while higher cytoplasmic *TLR2* expression is observed within smaller tumors. Older age contributes to upregulation of nuclear *TLR4*, a lipopolysaccharide receptor, as well as decreased expression of *TLR7*, which is a single-stranded RNA receptor [173,174].

### 5.11. Chromosomal Abnormalities

The presence of chromosomal abnormalities is correlated with a larger size of MCC and increased risk of metastases. They are more frequently observed within virus-negative than virus-positive MCCs. The median number of extensive genetic aberrations was 5.5 ± 1.1 changes per tumor. The majority of alterations are gains of chromosome arms as well as whole chromosomes [175]. Amplifications were observed on the following chromosomes: 1 (37–63%), 3q (33%), 5p (32–38%), 6p (42%), 8q (38%), 19 (63%), and X (41%), while losses are detected on 3p (46%), 4q (16%), 5q (21–26%), 7q (37,5%), 8p (21%), 10 (33%), 11q (17%), 13q (21–33%), 16q (11%), and 17p (25%). 13q14–21 and 13q12–12 deletion is associated with loss of the *RB1* and *BRCA2* gene, and the gain of 1p34 is correlated with overexpression of the *MYCL* gene [14,175,176,177,178,179].

### 5.12. MicroRNAs

miRNA profile varies between virus-positive and virus-negative MCCs. The expression profile of miR-203, miR-30a-3p, miR-769-5p, miR-34a, miR-30a-5p, and miR-375 significantly differs between those two types of MCC. Based on miRNA expression, MCCs could be divided into three groups, suggesting their heterogeneity, whereas MCPyV- MCCs are more diverse than MCPyV+ tumors. miR-203, which suppresses cell growth and inhibits proliferation by targeting oncogenic molecules, is overexpressed in MCPyV- MCCs, while it is expressed at a lower level in MCPyV+ MCCs. Thus, miR-203 is thought to promote regression of virus-negative MCC. Additionally, an increased level of miR-105 is considered to correlate with MCC metastases [177,180].

### 5.13. Other Genes

Loss-of-function mutations occur in *NF1*, which inhibits cell growth and turns off the RAS protein (which has an adverse, stimulating effect) as well as in *PRUNE2*, which is a proapoptotic factor. *PRUNE2* is suppressed in 62.5% of MCC cases [13]. The *GRIN2A* gene, which encodes a protein that forms a subunit of NMDA receptors, and *BRCA2*, which is involved in repairing damaged DNA, are downregulated in MCCs. The expression of the *TSC1* gene is also decreased [9,13]. Overexpression of matrix metalloproteinases (MMP), such as MMP7 and MMP10/2, is significantly associated with metastatic MCC (mMCC) [158]. The p16(INK4A) and p14(ARF) proteins are encoded by the cyclin-dependent kinase inhibitor 2A (*CDKN2A*) gene located at the short arm of chromosome 9 (9p21.3). Both of them control the cell cycle and are involved in cell senescence. The p16(INK4A) protein inhibits CDK4 and CDK6, which normally take part in cell cycle progression. p14(ARF) prevents p53 from being degraded. p16(INK4A) is upregulated in almost every MCC (97.7%) to prevent uncontrollable proliferation, resulting from suppression of *RB1* gene [166]. Mutations in DNA repair gene pathways, including mutL homolog 1 (*MLH1*), *ATM*, BRCA1 associated protein 1 (*BAP1*), checkpoint kinase 2 (*CHEK2*), and FA complementation group A (*FANCA*), were observed in 29% of MCC patients [13,181].

## 6. MCC Treatment

### 6.1. General Rules for MCC Treatment and Prognosis

MCC should be treated in highly specialized centers by an experienced multidisciplinary team. Surgical treatment is the mainstay of therapy in local and loco-regional primary MCC. For local disease, excision should be done with 1–2 cm margins and down to the fascia or periosteum. Patients with the clinical node-positive disease should undergo complete lymph node dissection (CLND). For clinical node-negative cases, sentinel lymph node biopsy (SLNB) is required. In some cases, CLND should be followed by radiation therapy [182]. Chemotherapy is used in some cases in the perioperative setting and unresectable and metastatic disease. Adjuvant chemotherapy for patients with stage I–III disease does not improve overall survival. Neoadjuvant and adjuvant chemotherapy are not routinely recommended for the loco-regional disease but may be considered in some selected cases. Based on recently published clinical trial results, immunotherapy has become the mainstay of treatment of metastatic disease. Chemotherapy, which was the standard of care in mMCC in the past, is recommended in case of contraindications to immunotherapy. Participation in clinical trials remains a valuable and recommended option [183].

MCC has a high rate of local recurrence, regional lymph node metastasis, and distant metastasis. The prognosis is poor and correlates with the stage of the disease. 10-year overall survival is about 65% in women and 50.5% in men (57% on average for all patients). The most important prognostic factor is the size of the primary tumor. Additionally, extracutaneous extension is of important prognostic value. Depending on the size of the primary MCC, the 10-year survival rate is 61% for tumors with up to 2 cm and 39% for tumors above 2 cm [184]. For patients with loco-regional disease (stage IIIB), the 5-year survival rate is 37%, and for patients with mMMC, it is 16% [185]. Lymph node involvement is also associated with poorer prognosis, with worsening of prognosis with increasing nodal involvement. Additionally, non-surgical treatment of the primary tumor is associated with worse outcomes, and positive or narrow surgical margins, lymphovascular invasion, and location in the head or neck are associated with poorer prognosis [182,186,187]. Comorbidities like HIV, chronic lymphocytic leukemia (CLL), solid organ transplant, and chronic T-cell immunosuppression are also known risk factors [182].

### 6.2. MCC Staging

The appropriate MCC management requires proper staging. MCC staging is performed according to the 8th edition of AJCC classification (American Joint Commission on Cancer). This system is based on the analysis of prognostic factors from 9387 MCC cases in the US [188,189,190].

### 6.3. Treatment of MCC Stage I–II

The goal of surgical treatment is complete surgical excision with clear surgical margins. Surgical margins have to be balanced with the morbidity of the surgery. The current guidelines include wide (to a margin of at least 1–2 cm) definitive scar excision. In some cases, when tissue sparing is necessary, Mohs micrographic surgery and modified Mohs surgery or other methods may be considered. If radiation therapy is planned, narrow surgical margins are likely sufficient. In the absence of detectable metastases to regional lymph nodes, a sentinel lymph node biopsy (SLNB) is recommended. The sentinel lymph node metastases occur in 25–35% of patients with no clinically detected metastases. The risk of micrometastases increases significantly in patients with a primary focus greater than 1 cm in diameter [182,191,192]. For patients with local or loco-regional disease who are not candidates for surgical treatment or refuse surgery, treatment including definitive radiotherapy or radiochemotherapy may be an acceptable option.

### 6.4. Treatment of MCC Stage III

**Figure 7:**
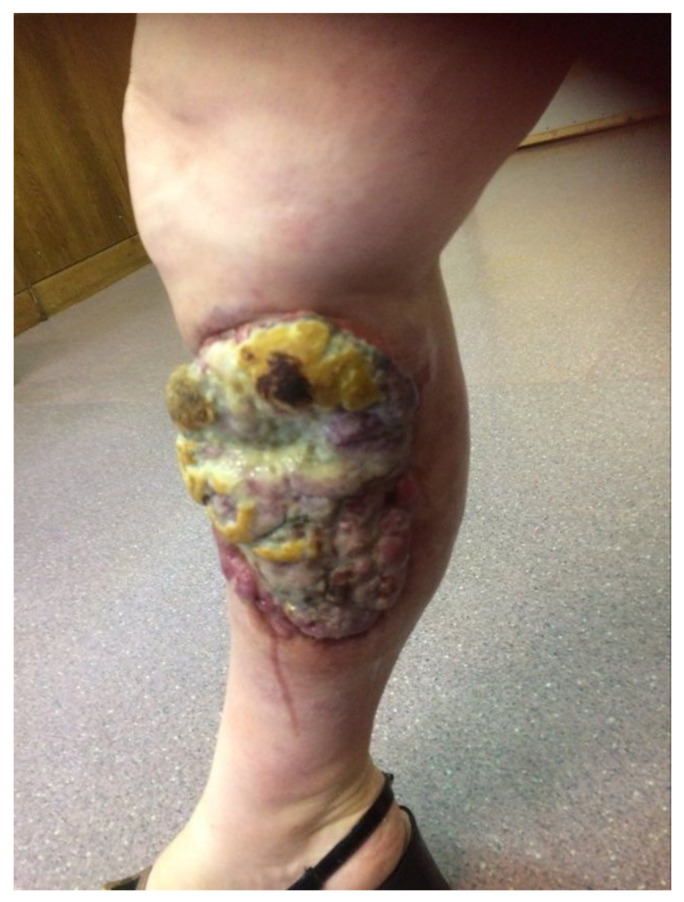
Locally advanced, unresectable MCC with in-transit metastases (stage III) in immunocompromised patient (patient with CLL, chronic lymphocytic leukemia).

In cases of the presence of metastases to regional lymph nodes (both micro-and macrometastases; stage III, Figure 7), complete lymph node dissection (CLND) is indicated. Adjuvant radiotherapy to the bed after lymph node dissection may improve treatment outcomes [193]. In some selected cases, perioperative systemic chemotherapy may be considered, but data about the efficacy of this approach are limited. No survival benefit has been reported in retrospective studies [194,195,196].

Even though the preliminary results of the trials with immunotherapy in the neoadjuvant setting are promising, this systemic treatment should not be used outside of clinical trials. The activity of nivolumab in the neoadjuvant setting has been shown in the CheckMate 358 study (NCT02488759). In this phase I/II clinical trial, thirty-nine patients with stage IA-IV MCC received nivolumab in a dose of 240 mg intravenously on days 1 and 15. The surgery was planned for day 29. The responses were assessed by imaging and pathology examinations. The surgery was done on thirty-six patients. Three patients were not operated on, one due to disease progression and two due to toxicity. A total of 47.2% of patients (*n* = 17) who underwent surgery achieved the pathological complete response (pCR). In 54.5% of thirty-three operated patients evaluable for radiological response (*n* = 18), tumor reductions of at least 30% were reported. Responses were not dependent on the status of TMB, PD-L1, and MCPyV. Tumor recurrence was not observed in patients with pCR. The median follow-up was 20.3 months. The median recurrence-free survival (RFS) and overall survival (OS) were not achieved. There was a significant correlation between RFS and pCR and radiological response at the time of surgery. Any grade treatment-related adverse events (TRAE) were reported in 46.2% of patients (*n* = 18), and TRAEs G3/G4 were observed in 7.7% of patients (*n* = 3). In this group of patients, no unexpected toxic effects were reported [197].

### 6.5. Radiotherapy

MCC is considered a radiosensitive tumor. Adjuvant radiotherapy in MCC could eliminate micrometastases and occult disease. However, there is a lack of evidence coming from randomized controlled trials. In a large retrospective analysis of 6908 patients with MCC, adjuvant radiotherapy significantly improved overall survival in patients without lymph node metastases (stage I and II) in comparison to non-irradiated patients [198]. Nevertheless, the same analysis showed that adjuvant radiotherapy did not improve the survival of patients with stage III disease. Other retrospective studies confirmed the benefit of adjuvant radiotherapy for local control and survival [193,199,200,201]. Thus, adjuvant radiotherapy should be considered in the majority of patients with stage I-III MCC after surgery, especially those with a primary tumor larger than 2 cm, inadequate surgical margins, lymphovascular space invasion, primary site in head and neck, or immunodeficiency [182]. There are no clear recommendations regarding target volumes, but in the vast majority of reports and protocols, elective margins are extensive, achieving 5 cm added to the primary tumor bed volume [182,202,203]. Moreover, it is recommended to consider encompassing the draining lymph node stations localized within 20 cm of the primary tumor [195,196]. The decision of elective lymph node irradiation should be based on nodal status. Adjuvant radiotherapy may be omitted in the case of negative SLNB in anatomical areas that are not at risk of false-negative results or after lymphadenectomy in the case of a single macroscopic clinically detected lymph node without extracapsular extension. The clear indications for nodal irradiation are clinically evident lymphadenopathy or positive SNLB without lymphadenectomy and multiple lymph node metastases and/or extracapsular extension after lymphadenectomy.

**Figure 8:**
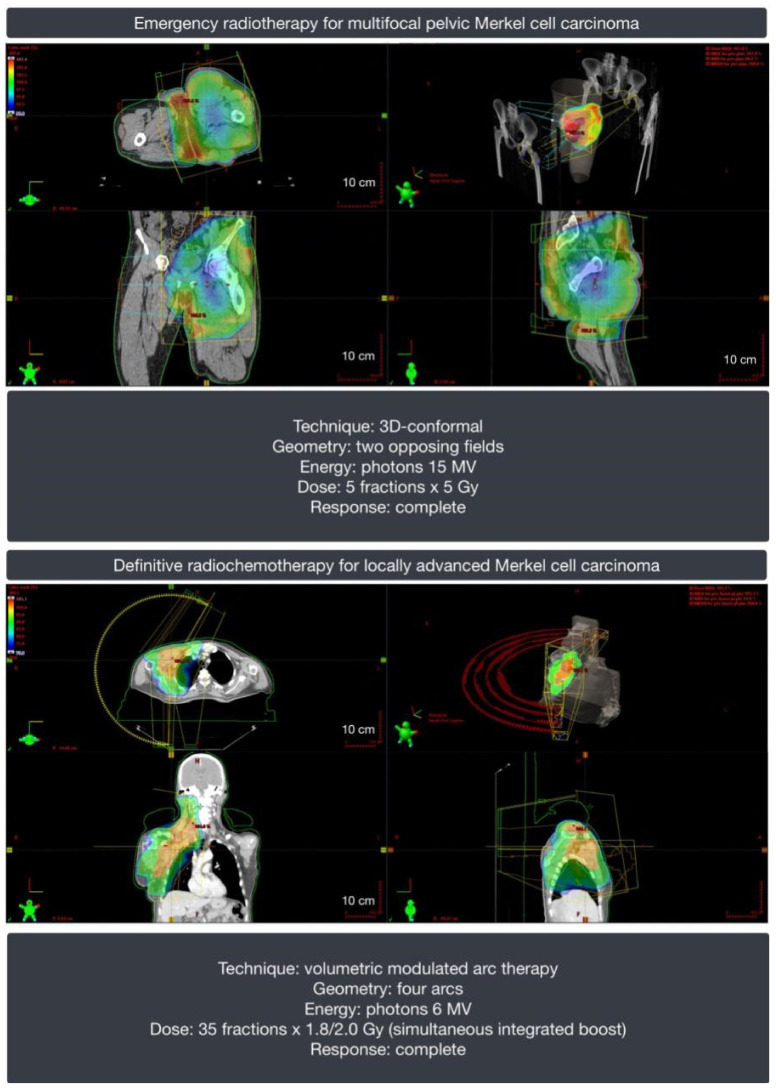
Radiotherapy plans for Merkell cell carcinoma.

Due to the high radiosensitivity of MCC, definitive radiotherapy or radiochemotherapy may be offered to the patients who are not suitable for surgery or refuse operation (Figure 8). There is no evidence regarding the routine use of radiochemotherapy both in the adjuvant and definitive settings. There are no clinical trials on the recommended fractionation regimens in MCC. The proposed total doses in 2 Gy fractions were proposed by NCCN (Table 1) [182].

### 6.6. Follow-Up after Definitive Treatment

Follow-up after loco-regional treatment in patients with MCC should include a complete physical examination, including skin and imaging examinations for distant metastases (especially for high-risk patients), and these should be done every 3–6 months for the first 3 years [182].

### 6.7. Treatment of Local and Disseminated Recurrences

**Figure 9:**
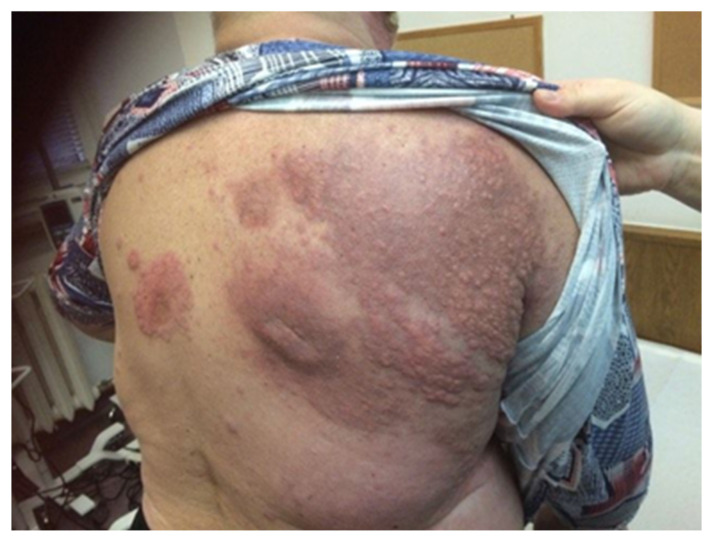
Extensive MCC skin lesions in patient with disease disseminated to skin and lungs (stage IV).

**Figure 10:**
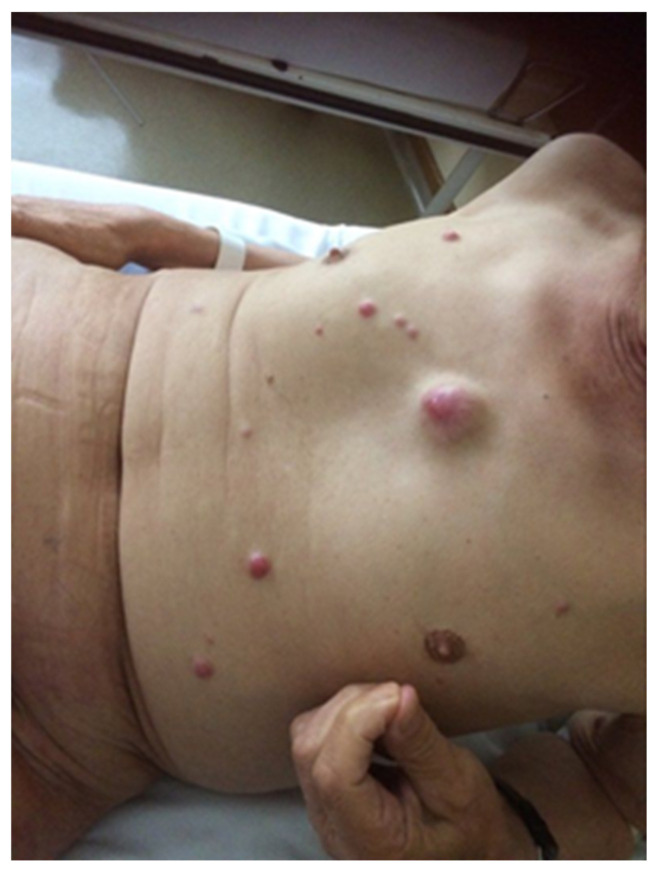
MCC spreading to the skin of the chest and abdomen (stage IV).

Published meta-analyses and retrospective analyses have shown that the prognosis in case of disease recurrence is poor. Local relapses are the most common and happen in about 30% of patients treated surgically. Postoperative radiation therapy reduces this percentage to approximately 11% [204]. Local recurrences can be treated as a primary MCC and should be individualized. If possible, tumor lesions should be excised with clear surgical margins, with adjuvant radiotherapy, if not used previously. Systemic chemotherapy may also be considered. For disseminated recurrences (Figure 9 and Figure 10), systemic therapy recommended for mMCC should be used [182].

### 6.8. Treatment of MCC Stage IV

#### 6.8.1. Chemotherapy

Historically based on similarities to small cell carcinoma, regimens of platinum and etoposide or cyclophosphamide, doxorubicin, and vincristine are most commonly used for first-line chemotherapy in MCC. Cyclophosphamide with doxorubicin or epirubicin and with vincristine combination was the most commonly used chemotherapy regimen with an overall response rate of 75.7% (35.1% complete, 35.1% partial, and 5.4% minor responses). Etoposide wth cisplatin or carboplatin was the next most commonly used regimen with an overall response rate of 60% (36% complete and 24% partial responses) [205]. In general, chemoregimens combining carboplatin, cisplatin, and etoposide, cyclophosphamide with vincristine, doxorubicin, prednisone, bleomycin, or 5-fluorouracil have been used in MCC treatment [206]. Most commonly cisplatin 25 mg/m^2^/day × 3 days (days 1 to 3) with etoposide 100 mg/m^2^/day × 3 days (days 1 to 3) is used. The high RR is not followed by durable responses in MCC. Tumors often recur/progress within 4–15 months [183]. A retrospective study of 6908 patients found that chemotherapy is not associated with an overall survival benefit in patients who presented with either local or nodal MCC [198,207]. In specific settings, chemotherapy is used. In a first-line setting, RR range from 53% to 61% with a median PFS of 3.1 months and duration of response < 8 months. In a second-line-setting, RR rates range from 23% to 45% with a median PFS of 2 months and duration of response <8 months. In a later-lines setting, RR range from 10% to 29% with no complete responses and median duration of response <2 months and median PFS of 2–3 months and OS of 4–5 months [5,208].

#### 6.8.2. Immunotherapy

One of the most recent and rapidly developing treatment options for all cancers, including MCC, is immunotherapy. Clinical studies with immune checkpoint inhibitors have demonstrated the clinical activity of anti-PDL1/anti-PDL1 antibodies in MCC. Additionally, durable responses have been shown. Due to the high activity of anti-PD-1 and anti-PD-L1 immune checkpoint inhibitors in the treatment of metastatic MCC, confirmed in phase II clinical trials, the current guidelines recommend immunotherapy as the treatment of choice for metastatic MCC. Additionally, pembrolizumab may be considered for recurrent regional disease [182]. 

In 2017, the FDA approved avelumab to treat adults and pediatric patients 12 years and older with metastatic Merkel cell carcinoma (MCC) irrespective of prior therapy, and EMA approved avelumab as monotherapy for the treatment of adult patients with metastatic Merkel cell carcinoma. These approvals were based on data from an open-label, single-arm, multicenter clinical trial JAVELIN Merkel 200 (NCT02155647). This study consisted of part A, which included patients treated in the second line (*n* = 88), and part B for systemic treatment-naive patients (*n* = 116). The first data from part A of this study, published in 2016, resulted in the approval of this drug for MCC therapy [209]. 

The patients eligible for part A of the JAVELIN Merkel 200 study were adult patients, in good performance status (ECOG 0-1, Eastern Cooperative Oncology Group 0-1), with mMCC confirmed by histology, disease progression following at least one previous systemic therapy used in the metastatic setting, measurable disease according to RECIST v. 1.1 criteria (Response Evaluation Criteria in Solid Tumors), and adequate bone marrow, renal, and hepatic function. The participants received avelumab at a dose of 10 mg/kg of body weight intravenously every 2 weeks until disease progression or unacceptable toxicity. The primary end-point was confirmed objective response (CR or PR) based on independent assessment and RECIST 1.1 criteria. Efficacy and safety populations included patients who received at least one dose of the study drug. The ORR was 31.8% (95% CI: 21.9–43.1%; *n* = 28), with CR in eight patients and PR in twenty patients. Stable disease (SD) was observed in nine patients. The responses were long-lasting and, at the time of analysis, were maintained in 23 patients. The duration of response (DOR) was at least 6 months in 92% of cases. The mPFS was 2.7 months (95% CI: 1.4–6.9), and the rate of PFS at 6 months was 40%. The PFS curve reached a plateau. The mOS was 11.3 months (95% CI: 7.5–14.0), and the OS rate at 6 months was 69%. In this analysis, five grade 3 treatment-related adverse events were reported in four (5%) patients: lymphopenia in two patients, aminotransferase increase in one patient, creatine phosphokinase increase in one patient, and blood cholesterol increase in one patient. No treatment-related Grade 4 AEs or treatment-related deaths were reported. In 74 patients, PD-L1 expression was assessed (≥1% positive cells), and it was present in 58 cases. The test for MCPyV was done in 77 cases and was positive in 60% (*n* = 46). Better outcomes were reported in patients who received fewer prior lines of systemic therapy [209]. The results from the first analysis were confirmed by the updated analyses with a median follow-up of 18 months and 24 months published in 2018. The patients were followed up for 29.2 months (24.8–38.1). The mOS was 12.6 months (95% CI: 7.5–17.1), and the 2-year survival rate was 36%. The median treatment duration was 3.9 months (0.5–36.3). The confirmed ORR was 33.0% (95% CI: 23.3–43.8). The median DOR was not reached (2.8–31.8). The PFS values were 29% after 12, 29% after 18, and 26% after 24 months of follow-up. Clinical activity was observed irrespectively of PD-L1 expression status and MCPyV status [210,211]. The results of the next updated analysis published in 2020 also confirmed the efficacy of avelumab in the group of previously pretreated patients. The ORR was 33.0% (95% CI: 23.3–43.8%). CR was observed in 10 patients (11.4%). In 17 of 29 patients who achieved response to treatment (58.6%), the response was maintained. Four patients had a continuous response lasting at least 3 years. DOR was 40.5 months (median; 95% CI: 18.0 months—not estimable). PFS rate at 2 years and 3 years was 26% (95% CI: 17–36%) and 21% (95% CI: 12–32%), respectively. After ≥ 44 months of follow-up, OS was 12.6 months (median; 95% CI: 7.5–17.1 months). OS rate at 3 years and 3.5 years were 32% (95% CI: 23–42%) and 31% (95% CI: 22–41%), respectively. High tumor mutational burden and high expression of MHC I (major histocompatibility complex class I) were associated with trends in the improvement of OS and ORR. Long-term responses, i.e., responses for at least 3 years, were observed regardless of PD-L1 expression. Any grade AEs and Grade ≥3 AEs were reported in 97.7% and 73.9%, respectively. Any grade TRAEs and TRAES G ≥3 occurred in 77.3% and 11.4% of participants, respectively. The most frequently reported TRAEs were fatigue, diarrhea, and nausea. Immune-related adverse events (irAE) were reported in nineteen patients (21.6%). Four irAE were Grade ≥3: increased transaminases, increased alanine aminotransferase, autoimmune disorder, and hypothyroidism. Eight patients (9.1%) discontinued therapy due to TRAEs. There were no deaths related to the study treatment [212].

The preliminary results of part B of JAVELIN Merkel 200 with avelumab in the first-line treatment of patients with mMCC were published in 2017. In 16 patients, after a follow-up period of at least 3 months, the unconfirmed response rate was 68.8% (95% CI: 41.3–89.0). Safety was assessed in 29 patients. TRAEs Grade ≥3 were reported in five patients (17.2%). They included two cases of infusion-related reaction, one case of aspartate aminotransferase increase, one case of alanine aminotransferase increase, one case of cholangitis, and one case of paraneoplastic syndrome. They were the reason for treatment discontinuation [213]. The extrapolated survival data were published in 2018. The mean survival rate was 49.9 months (6.3; 179.4), and 1-year and 5-year survival rates were 66% and 23%, respectively [214]. The updated results confirmed that 77.8% (14 out of 18) of treatment responses were maintained. The response duration in 83% of cases was longer than 6 months (95% CI: 46–96%) [215]. The next updated analysis, with a median follow-up of 21.2 months (range, 14.9–36.6), was published in 2019 (*n* = 116). The median treatment duration was 5.5 months (range, 0.5–35.4). Treatment was continued in 26 patients (22.4%) at the data cut-off. The ORR was 39.7% (95% CI: 30.7–49.2%), with CR in 19 patients (16.4%) and PR in 27 participants (23.3%). In PD-L1+ patients (*n* = 21), ORR was 61.9% (95% CI: 38.4–81.9%), and in the PD-L1- participants (*n* = 87), the ORR was 33.3% (95% CI: 23.6–44.3%). Median DOR was 18.2 months (95% CI: 11.3 months—not estimable). A response that lasted at least 6 months was seen in 35 patients. PFS rate at 6 months and at 12 months was 41% (95% CI: 32–50%) and 31% (95% CI: 23–40%), respectively. Median OS was 20.3 months (95% CI: 12.4 months—not evaluable). The OS rate at 1 year was 60% (95% CI: 50–68%), and in PD-L1+ and PD-L1 groups, 1 year OS rates were 71% (95% CI: 47–86%) and 56% (95% CI: 45–66%), respectively. Any grade TRAEs occurred in 94 patients (81.0%). Grade ≥3 TRAEs were reported in 21 participants (18.1%). No treatment-related deaths were reported [216].

The efficacy of avelumab in the real world was assessed in the expanded access program, which included mMCC patients with disease progression during or after chemotherapy and patients ineligible for chemotherapy or clinical trial participation. The efficacy and safety results were consistent with those from the JAVELIN Merkel 200 clinical trial (Table 2). The enrolled population also included patients who had an ECOG PS 2 or 3, had brain metastases stable after therapy, or were potentially immunocompromised. The median duration of avelumab treatment was 7.9 months (range, 1.0–41.7). A total of 240 out of 494 enrolled patients were evaluable for efficacy. The ORR was 46.7% in the evaluable patients, and 22.9% and 23.8% of participants achieved CR and PR. The safety data are limited. The most frequently reported AEs were an infusion-related reaction, fever, fatigue, rash, asthenia, abdominal pain, chills, and dyspnea [217].

Pembrolizumab, another immune checkpoint inhibitor, anti-PD1 monoclonal antibody, was approved by the FDA in 2018 for the treatment of patients with recurrent locally advanced or metastatic MCC, based on KEYNOTE-017 study results (NCT02267603). The primary end-point was ORR in the first-line setting. Fifty patients with MCC stage IIIB–IVC were enrolled in this study. The median follow-up was 14.9 months (range 0.4–36.4 months). The ORR was 56% (95% CI: 41.3–70.0%), with complete responses in 24% and partial responses in 32% of participants. Among the 28 patients with responses, a response duration of more than 6 months and 12 months was observed in 96% and 54% of participants, respectively. The median PFS was 16.8 months, and the PFS ratio after 2 years was 48.3%. The median OS was not reached, and the OS rate after 2 years was 68.7%. The presence of polyomavirus did not correlate with ORR, PFS, or OS. The ORR in MCPyV(+) and MCPyV(-) patients was 59% and 53%, respectively. In patients with PD-L1 expression, some trends for better PFS and OS were observed. Grade 1 or 2 treatment-related adverse events were reported in 68% of participants (*n* = 34). At least Grade 3 treatment-related adverse events occurred in 28% of 50 patients (*n* = 14) and led to treatment discontinuation in 14% of 50 patients (*n* = 7). One treatment-related death was reported in this study [218,219]. The safety and efficacy of nivolumab have been assessed in advanced MCC in a group of 25 patients in CheckMate 358 study (Table 2). The ORR rate in patients evaluable for response (*n* = 22) was 68% after the 26-week follow-up (range 5–35 weeks), 71% (*n* = 14) in treatment-naïve participants, and 65% in pretreated patients (*n* = 8) [220].

#### 6.8.3. The Resistance to the Available Systemic Therapies

**Figure 11:**
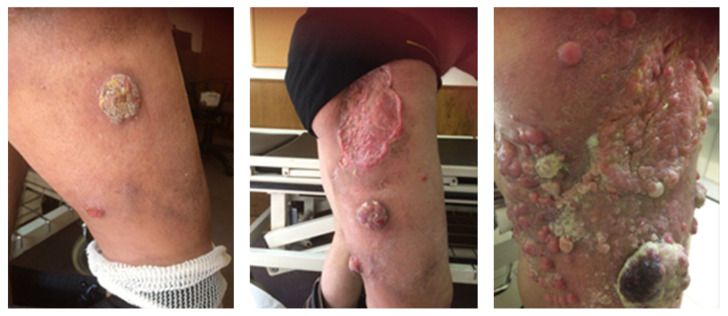
Progressive MCC despite of few lines of systemic therapies, inluding immunotherapy.

MCC resistance, either primary or secondary, to chemotherapy and checkpoint inhibitors remains a major clinical challenge (Figure 11). The data regarding effective therapy for patients whose disease is refractory to PD-1 pathway blockade are missing. The published case studies and case series describe patients treated with additional immune checkpoint inhibitors after MCC progression during anti-PD-1 therapy. LoPiccolo et al. [116] reported objective responses in four of thirteen (31%) patients treated with anti-CTLA-4, alone or in combination with anti-PD-1. There are currently many clinical trials conducted to evaluate the effectiveness of new therapies in MCC. 

In fact, 28 clinical trials for Merkel cell carcinoma were found using the following keywords: Merkel cell carcinoma, solid tumors on 25th April 2021 (Table 3). They were registered on https://clinicaltrials.gov and https://www.clinicaltrialsregister.eu/ (Accessed on 25 April 2021).

## 7. Materials and Methods

Pubmed/MEDLINE and Google Scholar databases were used in the process of writing the review. The searched keywords were MCC, MCPyV, immune evasion, PD-L1, mutational burden, TP53, RB1, treatment, and immunotherapy. The authors used NCCN guildlines and AJCC classification. Clinical trials for Merkel cell carcinoma, which were registered on the https://clinicaltrials.gov and https://www.clinicaltrialsregister.eu/ websites, were found using the following keywords: Merkel cell carcinoma and solid tumors on April 25th 2021. The graphics were created using Google Drawings. All the photos were from authors’ private archives.

## 8. Conclusions

Merkel cell carcinoma is an uncommon and highly aggressive skin cancer developing within the dermis and subcutis with an immunophenotype corresponding to sensory Merkel cells of the skin—mechanoreceptor cells of the basal layer of the epidermis. Historically, advanced MCC patients have a poor prognosis, with less than 20% alive at five years. MCC is transiently sensitive to chemotherapy, including platinum agents and etoposide (EP chemo regimen), with reported first-line response rates (RR) between 53% to 61%. In March 2017, the first approval of immunotherapy, avelumab—an anti-PD-L1 checkpoint inhibitor—has increased the PFS of MCC patients. In the avelumab trial, 74% of responses were longer than one year (median duration not reached). Thanks to the development of next-generation sequencing (NGS), the mutational profile of MCC was recently described in detail. It was shown that the pathogenesis of MCC is variable with MCPyV integrated within the genome detected in 60–80% of cases. The mutational tumor burden of Merkel cell carcinoma varies significantly between virus-negative and virus-positive MCCs. Viral negative MCCs are characterized by the UV damage signature—involving C to T transitions at dipyrimidine bases (i.e., CC to TT). In particular, mutations occur in *RB1*, *TP53*, and *NOTCH* genes as well as in the PI3K–AKT–mTOR and HH pathways. The PI3K–AKT–mTOR pathway is often hyperactivated in MCCs, and p16(INK4A) is upregulated in almost every MCC. Viral-negative tumors show low to non-expression of RB protein deriving from *RB1* copy number loss and loss-of-function of the gene. Similarly, pathogenic mutations in the *TP53* gene and deregulation of p53 protein expression are also detected mostly in viral-negative tumors. The presence of chromosomal abnormalities is correlated with a larger size of MCC and an increased risk of metastases. They are more frequently observed within virus-negative than virus-positive MCCs. Genome-wide copy number analysis has confirmed recurrent large regions of chromosomal gain and loss. Similarly, the expression profile of miR-203, miR-30a-3p, miR-769-5p, miR-34a, miR-30a-5p, and miR-375 significantly differs between those two types of MCC. Recent data on the molecular biology of MCC enable the identification of new potential therapeutic targets. Currently, multiple clinical trials investigating novel drugs, including immunotherapy, are ongoing. MCC patients should be treated by an experienced multidisciplinary team.

## Figures and Tables

**Table 1:** Total doses in 2 Gy fractions recommended by National Comprehensive Cancer Network Guidelines in various clinical situations.

Setting	Indication	Dose Range
Adjuvant	Negative resection margins, high-risk	50–56 Gy
	Microscopically positive resection margins	56–60 Gy
	Macroscopically positive resection margins	60–66 Gy
	Positive sentinel lymph node biopsy with no evidence of clinically palpable or radiologically suspicious for nodal metastases without lymphadenectomy	50–56 Gy
	Multiple involved lymph nodes and/or extracapsular extension after lymphadenectomy	56–60 Gy
Definitive	Unresectable or refused surgery or at high risk of postsurgical morbidity	60–66 Gy
	Clinically palpable or radiologically evident nodal metastases without lymphadenectomy	60–66 Gy

**Table 2:** The summary of anti-PD-L1/anti-PD-1 antibodies efficacy in advanced/metastatic MCC.

Molecule	Target	Study Name	Study Number	Number of Patients	Treatment Line	ORR	mPFS	mOS
avelumab	PD-L1	JAVELIN Merkel 200	NCT02155647	116	1	39.7%	4.1 months	20.3 months
avelumab	PD-L1	JAVELIN Merkel 200	NCT02155647	88	>1	33%	2.7 months	12.6 months
pembrolizumab	PD-1	Keynote-017	NCT02267603	50	1	56%	16.8 months	NR
nivolumab	PD-1	CheckMate-358	NCT02488759	14	1	71%	NR	NR
nivolumab	PD-1	CheckMate-358	NCT02488759	8	>1	63%	NR	NR

NR, not reached.

**Table 3:** Current ongoing and recruting clinical trials for Merkel cell carcinoma (April 2021).

Clinical Trial	Agent/Interventions	Phase	Study Population	Status
**NCT04792073**	Avelumab Comprehensive Ablative Radiation Therapy	Phase 2	Merkel Cell Carcinoma	Recruiting
**NCT03599713**	Retifanlimab	Phase 2	Merkel Cell Carcinoma	Recruiting
**NCT04393753**	Domatinostat in combination with avelumab	Phase 2	Merkel Cell Carcinoma	Recruiting
**NCT03787602**	KRT-232	Phase 2	Merkel Cell Carcinoma	Recruiting
**NCT04261855**	Avelumab External Beam Radiation Therapy (EBRT) Lutetium-177 (177Lu)-DOTATATE	Phase 1 Phase 2	Merkel Cell Carcinoma	Recruiting
**NCT03853317**	Avelumab N-803 haNK™	Phase 2	Merkel Cell Carcinoma	Recruiting
**NCT03071406**	Nivolumab Ipilimumab Stereotactic Body Radiation Therapy (SBRT)	Phase 2	Merkel Cell Carcinoma	Recruiting
**NCT04590781**	XmAb18087 XmAb18087 ± Pembrolizumab	Phase 2	**Merkel Cell Carcinoma** Small Cell Lung Cancer	Not yet recruiting
**NCT03271372**	Avelumab	Phase 3	Merkel Cell Carcinoma	Recruiting
**NCT04160065**	IFx-Hu2.0	Phase 1	**Merkel Cell Carcinoma** Cutaneous Squamous Cell Carcinoma	Recruiting
**NCT04291885**	Avelumab Placebo	Phase 2	**Merkel Cell Carcinoma** Neuroendocrine Tumors Carcinoma Neuroendocrine Skin	Recruiting
**NCT03684785**	Cavrotolimod Pembrolizumab Cemiplimab	Phase 1 Phase 2	**Merkel Cell Carcinoma** Cutaneous Squamous Cell Carcinoma and other Solid Tumors	Recruiting
**NCT03712605**	Best Practice Pembrolizumab Radiation Therapy	Phase 3	Merkel Cell Carcinoma	Recruiting
**NCT03901573**	NT-I7 Atezolizumab	Phase 1 Phase 2	Melanoma **Merkel Cell Carcinoma** Cutaneous Squamous Cell Carcinoma	Recruiting
**NCT02978625**	Nivolumab Talimogene Laherparepvec	Phase 2	Cutaneous Squamous Cell Carcinoma **Merkel Cell** **Carcinoma** Other Rare Skin Tumors	Recruiting
**NCT03458117**	Talimogene Laherparepvec (T-VEC)	Phase 1	Non-melanoma Skin Cancer	Recruiting
**NCT03747484**	Autologous MCPyV-specific HLA-A02-restricted TCR-transduced CD4+ and CD8+ T-cells FH-MCVA2TCR Avelumab Pembrolizumab Fludarabine Cyclophosphamide	Phase 1 Phase 2	Merkel Cell Carcinoma	Recruiting
**NCT04725331**	BT-001 Pembrolizumab	Phase 1 Phase 2	Solid Tumor, Adult Metastatic Cancer Soft Tissue Sarcoma **Merkel Cell Carcinoma** Melanoma Triple Negative Breast Cancer Non Small Cell Lung Cancer	Recruiting
**NCT04246671**	TAEK-VAC-HerBy	Phase 1 Phase 2	Breast Cancer Gastric Cancer Chordoma Lung Cancer Ovarian Cancer Prostate Cancer Colorectal Cancer P ancreatic Cancer Hepatocellular Cancer **Merkel Cell Carcinoma** Small-cell Lung Cancer	Recruiting
**NCT03935893**	Tumor Infiltrating Lymphocytes (TIL) Fludarabine + Cyclophosphamide combination	Phase 2	**Merkel Cell Carcinoma** Advanced Solid Cancers	Recruiting
**NCT04116320**	Device: Echopulse Imiquimod Standard of Care PD-1 Therapy	Phase 1	**Merkel Cell Carcinoma** Advanced Solid Cancers	Recruiting
**NCT04272034**	INCB099318	Phase 1	**Merkel Cell Carcinoma** Advanced Solid Cancers	Not yet recruiting
**NCT04242199**	INCB099280	Phase 1	**Merkel Cell Carcinoma** Advanced Solid Cancers	Recruiting
**NCT04260802**	OC-001 OC-001 in Combination	Phase 1 Phase 2	**Merkel Cell Carcinoma** Advanced or Metastatic Cancers	Recruiting
**NCT03841110**	FT500 Nivolumab Pembrolizumab Atezolizumab Cyclophosphamide Fludarabine IL-2	Phase 1	**Merkel Cell Carcinoma** Advanced Solid Tumors	Recruiting
**NCT02643303**	Durvalumab Tremelimumab Poly ICLC	Phase 1 Phase 2	**Merkel Cell Carcinoma** Advanced, Measurable, Biopsy-accessible Cancers	Recruiting
**NCT03212404**	CK-301 (cosibelimab)	Phase 1	**Merkel Cell Carcinoma** Advanced Cancers	Recruiting
**NCT04551885**	FT516 Avelumab Cyclophosphamide Fludarabine Drug: IL-2	Phase 1	Advanced Solid Tumors	Recruiting

## Data Availability

No new data were created or analyzed in this study. Data sharing is not applicable to this article.
